# Oncogenic β-catenin stimulation of AKT2–CAD-mediated pyrimidine synthesis is targetable vulnerability in liver cancer

**DOI:** 10.1073/pnas.2202157119

**Published:** 2022-09-19

**Authors:** Fangming Liu, Xiaochen Gai, Yuting Wu, Baohui Zhang, Xiaoyu Wu, Rongrong Cheng, Bufu Tang, Kezhuo Shang, Na Zhao, Weiwei Deng, Jie Chen, Zhengyi Zhang, Song Gu, Liang Zheng, Hongbing Zhang

**Affiliations:** ^a^State Key Laboratory of Medical Molecular Biology, Haihe Laboratory of Cell Ecosystem, Department of Physiology, Institute of Basic Medical Sciences and School of Basic Medicine, Chinese Academy of Medical Sciences and Peking Union Medical College, Beijing 100005, China;; ^b^Department of Physiology, School of Life Science, China Medical University, Shenyang, Liaoning 110122, China;; ^c^Institute of Pediatric Translational Medicine, Shanghai Children’s Medical Center, Shanghai Jiao Tong School of Medicine, Shanghai 200127, China;; ^d^Department of Radiology, Sir Run Run Shaw Hospital, Zhejiang University School of Medicine, Hangzhou, Zhejiang 310016, China;; ^e^Department of Pathology, Peking Union Medical College Hospital, Chinese Academy of Medical Sciences and Peking Union Medical College, Beijing 100730, China;; ^f^Division of Cardiology, Department of Medicine, University of California, Los Angeles, CA 90095, USA;; ^g^Molecular Biology Institute, University of California, Los Angeles, CA 90095, USA;; ^h^Department of General Surgery/Surgical Oncology Center, Shanghai Children’s Medical Center, Shanghai Jiao Tong University School of Medicine, Shanghai 200127, China;; ^i^Department of Pharmacology and Chemical Biology, Shanghai Jiao Tong University School of Medicine, Shanghai 200025, China;; ^j^Fujian Branch of Shanghai Children's Medical Center, Shanghai Jiaotong University School of Medicine, Fujian Children's Hospital, Fuzhou, Fujian 350014, China

**Keywords:** β-catenin, CAD, AKT2, pyrimidine synthesis, liver cancer

## Abstract

*CTNNB1*, encoding β-catenin protein, is the most frequently altered proto-oncogene in hepatic neoplasms. In this study, we studied the significance and pathological mechanism of *CTNNB1* gain-of-function mutations in hepatocarcinogenesis. Activated β-catenin not only triggered hepatic tumorigenesis but also exacerbated *Tp53* deletion or hepatitis B virus infection–mediated liver cancer development in mouse models. Using untargeted metabolomic profiling, we identified boosted de novo pyrimidine synthesis as the major metabolic aberration in β-catenin mutant cell lines and livers. Oncogenic β-catenin transcriptionally stimulated AKT2, which then phosphorylated the rate-limiting de novo pyrimidine synthesis enzyme CAD (carbamoyl-phosphate synthetase 2, aspartate transcarbamoylase, dihydroorotase) on S1406 and S1859 to potentiate nucleotide synthesis. Moreover, inhibition of β-catenin/AKT2-stimulated pyrimidine synthesis axis preferentially repressed β-catenin mutant cell proliferation and tumor formation. Therefore, β-catenin active mutations are oncogenic in various preclinical liver cancer models. Stimulation of β-catenin/AKT2/CAD signaling cascade on pyrimidine synthesis is an essential and druggable vulnerability for β-catenin mutant liver cancer.

## Significance

β-Catenin encoding gene *CTNNB1* is known as the most frequently mutated proto-oncogene in liver cancer. We report that active β-catenin is essential in initiation and advancement of hepatocarcinogenesis. As a transcriptional activator of AKT2, β-catenin potentiates AKT2 phosphorylation of CAD, which in return stimulates de novo pyrimidine synthesis and liver cancer development. β-Catenin, AKT2, and pyrimidine synthesis inhibitors are promising therapeutics for the treatment of oncogenic β-catenin–associated cancer.

Liver cancer is the sixth most common cancer and the third leading cause of cancer death worldwide (1). It is the second most lethal tumor after pancreatic cancer (2). Hepatic cancer is a heterogenous group of malignances ranging from hepatocellular carcinoma (HCC) (75 to 85%), intrahepatic cholangiocarcinoma (ICC) (10 to 15%), to several rare subtypes, such as hepatoblastoma (HB), the most common pediatric liver cancer (3). Its multifaceted etiologies include genetic aberrations, hepatitis B virus (HBV) or hepatitis C virus (HCV) infection, and chemical carcinogen induction. These complex pathological events are still not druggable. The knowledge of the molecular events governing tumor initiation and progression may aid the development of targeted therapies. Therefore, genetic alterations should be studied for their impacts on hepatocarcinogenesis.

Cancer is a genetic disease, which usually arises from synergistic interaction between activated proto-oncogenes and inactivated tumor suppressors. *CTNNB1* (coding for β-catenin) (22% mutation rate) and *TP53* (29% mutation rate) are the most frequently altered proto-oncogenes and tumor suppressor genes, respectively, detected mainly in HCC (https://cancer.sanger.ac.uk/cosmic/browse/tissue?wgs=off&sn=liver&ss=all&hn=all&sh=all&in=t&src=tissue&all_data=n) (4). Over half of HBs also harbor *CTNNB1* mutations (5). In addition, *CTNNB1* mutation is identified in more than 10% hepatocellular adenoma (HCA), which is a benign liver neoplasm with risk of malignant transformation (6). Mutations of exon 3 are the most common alterations of β-catenin in liver cancer (7). Phosphorylation of serine/threonine encoded by exon 3 of *CTNNB1* leads to ubiquitination-mediated degradation of β-catenin. Exon 3 variations protect β-catenin protein from degradation and in return constitutively turn on β-catenin signaling cascade (8, 9). Although activated β-catenin signaling is required for hepatic *APC* knockout mice to develop liver cancer (10), mice did not developed liver tumors 6 m after hepatic *Ctnnb1* exon 3 deletion (9). Thus, whether altered β-catenin is the cause or the consequence of liver cancer is largely unknown.

More than half of global HCC cases are HBV positive and 70 to 80% of HCC patients in HBV endemic regions are HBV positive (11). Since up to 10% Chinese are HBV carriers, half of HCC patients are in China (12). Liver cancer is the fourth most common cancer and the third leading cause of cancer death in China (13). Chronic hepatitis caused by HBV is thought to be the major cause of HCC. Given that only a small fraction of HBV-infected people develop hepatic cancer, additional genomic insults from ingestion of foods tainted with chemical carcinogens such as aflatoxins and excessive consumption of alcohol may contribute to eventual development of liver cancer in HBV carriers. For instance, 13.4 to 19% of liver cancer patients infected with HBV harbor *CTNNB1* mutations in tumor lesions (14, 15), suggesting a potential collaboration of these two pathological events in liver cancer development.

Metabolic aberration is a hallmark of cancer (16). Oncogenic mutations of proto-oncogenes or tumor suppressors reprogram metabolism to meet enhanced nutritional and growth requirements of unchecked cell proliferation and tumor growth (17). Liver is the major metabolic organ. The relationship between abnormal metabolism and hepatic cancer remains largely elusive. If mutated β-catenin contributes to hepatocarcinogenesis, elucidation of its downstream events such as aberrant metabolism and its governing signaling transduction pathway may provide novel targets for the treatment of liver cancer.

To establish the causative relationship between aberrant β-catenin activation and liver cancer, we not only dissected the role of *CTNNB1* mutation alone in liver carcinogenesis but also demonstrated its collusion with *Tp53* deletion or transgenic *HBV* in liver cancer propagation. Moreover, we identified oncogenic β-catenin–boosted de novo pyrimidine synthesis via stimulation of the AKT2–CAD (carbamoyl-phosphate synthetase 2, aspartate transcarbamoylase, dihydroorotase) signaling pathway in liver cancer development and its clinical significance in cancer treatment.

## Results

### Constitutively Activated β-Catenin Causes Hepatic Tumors.

**Fig. 1.**
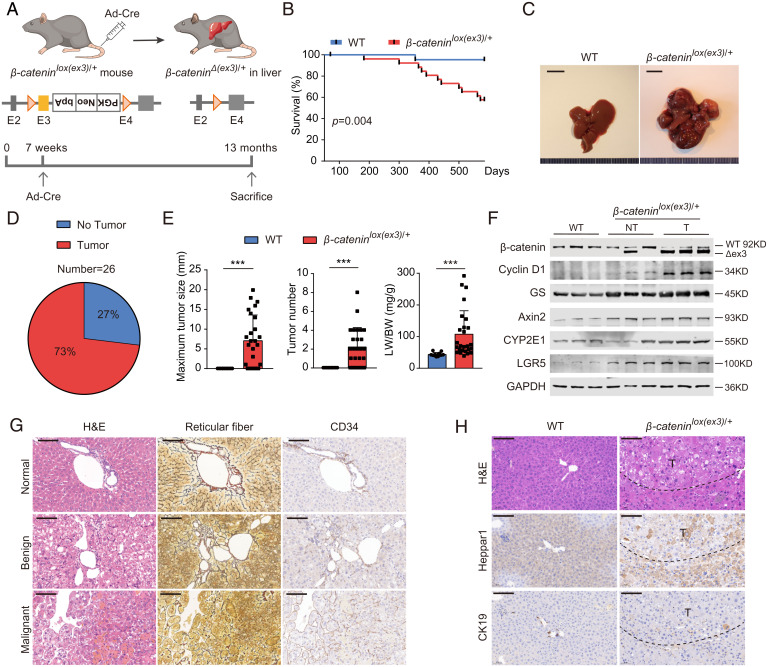
β-Catenin activation causes hepatic tumors. (*A*) Schematic illustration of *Cre*-adenovirus–induced β-catenin active mutation in mouse liver. (*B*) Survival of WT mice (*n* = 23) and *β-catenin^lox(ex3)/+^* mice (*n* = 27) 50 d post *Cre*-adenovirus tail vein injection. (*C*) Representative liver pictures of 13-mo-old mice. (*D*) Tumor incidence (at least one visible tumor nodule on the surface of liver) of 13-mo-old *β-catenin^lox(ex3)/+^* mice (*n* = 26). (*E*) The maximum liver tumor size (*Left*), tumor number (*Middle*), and ratio of liver weight to body weight (*Right*). WT mice (*n* = 12), *β-catenin^lox(ex3)/+^* mice (*n* = 26). Data are shown as mean ± SD. (*F*) Immunoblotting of mouse liver tissues. (*G*) Representative liver H&E, reticular fiber, and CD34 stainings of *β-catenin^lox(ex3)/+^* mice. (*H*) Immunohistochemistry stainings of mouse liver tissues. (Scale bar: 100 μm.) ***P < 0.001.

Because exon 3 is the most common locus of *CTNNB1* mutation in cancer (7), exon 3–deleted mice are widely used for the study of β-catenin activation (9). To mimic *CTNNB1* active mutations in hepatoblastoma and HCC, we first generated albumin (Alb)-Cre–mediated hepatic *Ctnnb1* exon 3–deleted mice. However, these mice died within 20 to 30 d after birth with hepatomegaly (*SI Appendix*, Fig. S1 *A–C*). Since early mouse lethality due to high efficacy of β-catenin activation precluded us from using this model to study hepatocarcinogenesis, we inoculated 7-wk-old *β-catenin^lox(ex3)/+^* mice with *Cre*-adenoviruses (6 × 10^8^ pfu per mice) via tail vein injection to generate mosaic *Ctnnb1* exon 3 deletion in mouse livers (Fig. 1*A*). A total of 20 to 40% of *β-catenin^lox(ex3)/+^* mice died within 50 to 100 d after *Cre*-adenovirus incubation (*SI Appendix*, Fig. S1 *D–F*) (9). The remaining *β-catenin^lox(ex3)/+^* mice also had a shorter lifespan albeit at a slower pace (Fig. 1*B*). By 13 mo old of age, 73% (19/26) of the survived *β-catenin^lox(ex3)/+^* mice but not wild-type (WT) mice developed prominent liver tumors with activated β-catenin (Fig. 1 *C–F*). Among 19 tumor-bearing *β-catenin^lox(ex3)/+^* mice, three had benign neoplasms, eight had malignant ones, and the remaining eight mice had both types of tumors according to hematoxylin-eosin (H&E), reticular fiber, and CD34 stainings (Fig. 1*G* and *SI Appendix*, Table S1). Malignant lesions had strongly diffused staining of CD34 and reticulin widening, while benign lesions showed minimal/focal positivity of CD34 and relative normal reticulin staining. Positive Heppar1 and negative CK19 stainings indicated that the malignant tumors were HCC rather than ICC (Fig. 1*H*). As it took a long latency for mutant β-catenin to cause liver cancer, additional pathological events may have participated in this pathological process.

### Oncogenic β-Catenin Collaborates with Distinct Carcinogenic Factors in the Promotion of Hepatocarcinogenesis.

**Fig. 2.**
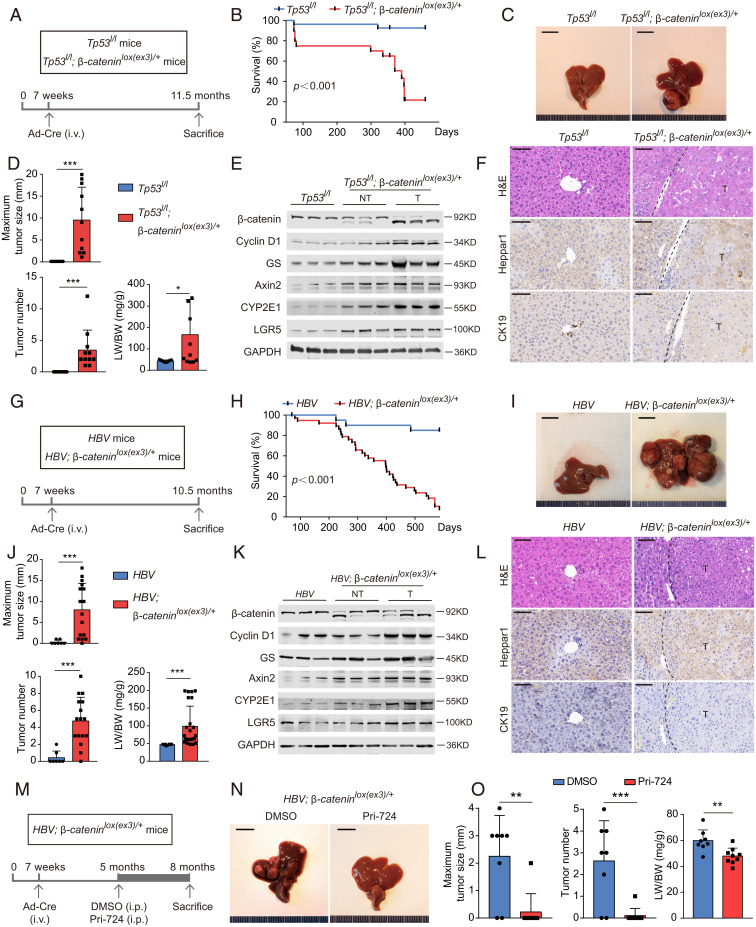
Oncogenic β-catenin colludes with distinct carcinogenic factors in furtherance of hepatocarcinogenesis. (*A–F*) β-Catenin activation and *Tp53* deletion in mice. (*A*) *Tp53^l/l^* and *Tp53^l/l^; β-catenin^lox(ex3)/+^* mice were injected with *Cre*-adenovirus via tail vein 7 wk after birth. (*B*) Survival of *Tp53^l/l^* mice (*n* = 27) and *Tp53^l/l^; β-catenin^lox(ex3)/+^* mice (*n* = 20). (*C–F*) Representative pictures (*C*), the maximum liver tumor size (*Upper*), tumor number (*Lower Left*), ratio of liver weight to body weight (*Lower Right*) (*D*), immunoblotting (*E*), and representative H&E, Heppar1 and CK19 stainings (*F*) of liver tissues from 11.5-mo-old mice. (*G–L*) Mutant β-catenin and transgenic HBV in mice. (*G*) *HBV* and *HBV; β-catenin^lox(ex3)/+^* mice were injected with *Cre*-adenovirus to generate *HBV* and *HBV* plus hepatic *β-catenin* mutant mice. (*H*) Survival of *HBV* mice (*n* = 21) and *HBV; β-catenin^lox(ex3)/+^* mice (*n* = 39). (*I–L*) Representative pictures (*I*), the maximum liver tumor size (*Upper*), tumor number (*Lower Left*), ratio of liver weight to body weight (*Lower Right*) (*J*), immunoblotting (*K*), and representative H&E, Heppar1 and CK19 stainings (*L*) of liver tissues from 10.5-mo-old mice. (*M–O*) Pri-724 treatment. (*M*) *HBV; β-catenin^lox(ex3)/+^* mice were injected with *Cre*-adenovirus by 7 wk, treated with vehicle (DMSO, dimethyl sulfoxide) or Pri-724 by 5 mo and killed by 8 mo after birth. (*N*) Representative liver pictures of 8-mo-old mice. (*O*) The maximum liver tumor size (*Left*), tumor number (*Middle*), and ratio of liver weight to body weight (*Right*) were analyzed. Data are shown as mean ± SD *P < 0.05, **P < 0.01, ***P < 0.001.

*CTNNB1* and *TP53* are the most commonly mutated proto-oncogenes and tumor suppressor genes in liver cancer. Guichard et al. reported that *CTNNB1* and *TP53* mutations were mutually exclusive, as only 2.4% (3/125) of HCC had co-occurrence of *CTNNB1* and *TP53* mutations, 11.5% (3/26) of *TP53* mutant HCC had *CTNNB1* mutations, and 7.3% (3/41) of *CTNNB1* mutant HCC had *TP53* mutation in that study (14). However, we found concomitant *CTNNB1* and *TP53* mutations in a bigger subset of liver cancers by analyzing the HCC sequencing data of 471 HCC cases compiled from several reports (15, 18–20). While only 7.5 to 11.3% of enrolled HCC had concurrent *CTNNB1* and *TP53* mutations and 10.2 to 20.2% of TP53 mutant HCC harbored *CTNNB1* mutations, half (46 to 53%) of *CTNNB1* mutant HCC carried *TP53* mutations (*SI Appendix*, Table S2) (15, 18–20), suggesting the critical role of *TP53* mutations in mutant *CTNNB1*-associated liver cancer, at least for HCC patients in China. To simulate this subtype of liver cancer, we crossed *Tp53^l/l^* mice with *β-catenin^lox(ex3)/+^* mice to generate *Tp53^l/l^; β-catenin^lox(ex3)/+^* mice. *Cre*-adenoviruses were then injected to mutate *Ctnnb1* and/or delete *Tp53* in liver accordingly (Fig. 2*A*). *Tp53^l/l^; β-catenin^lox(ex3)/+^* mice had shorter lifespans than *Tp53^l/l^* mice (Fig. 2*B*). *Tp53^l/l^; β-catenin^lox(ex3)/+^* mice but not *Tp53^l/l^* mice developed obvious liver tumors when they were 11.5 mo old (Fig. 2 *C* and *D*). These lesions were typical HCC with activated β-catenin (Fig. 2 *E* and *F*). We also generated heterozygous *Ctnnb1* exon 3–deleted mouse embryonic fibroblasts (MEFs) in *Tp53* null background from *Tp53^l/l^; β-catenin^lox(ex3)/+^* mouse embryos with addition of *Cre*-adenoviruses in cell culture (*SI Appendix*, Fig. S2*A*). Activated β-catenin and deleted TP53 MEFs exhibited stronger tumorigenic potential than TP53 null MEFs did in immunodeficient nude mice (*SI Appendix*, Fig. S2 *B–E*).

A total of 13.4 to 19% of liver cancer patients infected with HBV harbor *CTNNB1* mutations in tumor lesions (14, 15). To imitate this subset of HCC, we crossed *HBV* transgenic mice with *β-catenin^lox(ex3)/+^* mice to generate *HBV* mice and *HBV; β-catenin^lox(ex3)/+^* ones. These mice were then injected with *Cre*-adenoviruses to delete exon 3 of *Ctnnb1* in hepatocytes of *HBV; β-catenin^lox(ex3)/+^* mice (Fig. 2*G*). *HBV; β-catenin^lox(ex3)/+^* mice had a shorter lifespan compared to *HBV* transgenic mice (Fig. 2*H*) and developed HCC with activated β-catenin at age of 10.5 mo (Fig. 2 *I–L*).

Comparison of the survivals of various mouse models we generated revealed that *β-catenin^lox(ex3)/+^* mice appear to have shortened survival than that of *HBV* mice (*P* = 0.0645) or *Tp53^l/l^* mice (*P* = 0.0538) albeit without reaching statistical significance within the window of observation (*SI Appendix*, Fig. S3). Therefore, the relative potency of tumorigenic potential among β-catenin activation, transgenic HBV, and *Tp53* deletion in mice is inconclusive. Combination of either transgenic *HBV* and β-catenin activation or *Tp53* deletion and β-catenin activation was more potent than either transgenic *HBV*, β-catenin activation, or *Tp53* deletion alone in malignant transformation (*SI Appendix*, Fig. S3). Pri-724, a β-catenin inhibitor, blunted the tumor growth in *Cre*-adenovirus–inoculated *HBV; β-catenin^lox(ex3)/+^* mice without changes in mouse body weight (Fig. 2 *M–O* and *SI Appendix*, Fig. S4). Taken together, under various preclinical settings, oncogenic β-catenin promotes mouse hepatocarcinogenesis, which can be ameliorated by β-catenin inhibitor.

### Oncogenic β-Catenin Stimulates De Novo Pyrimidine Synthesis.

**Fig. 3.**
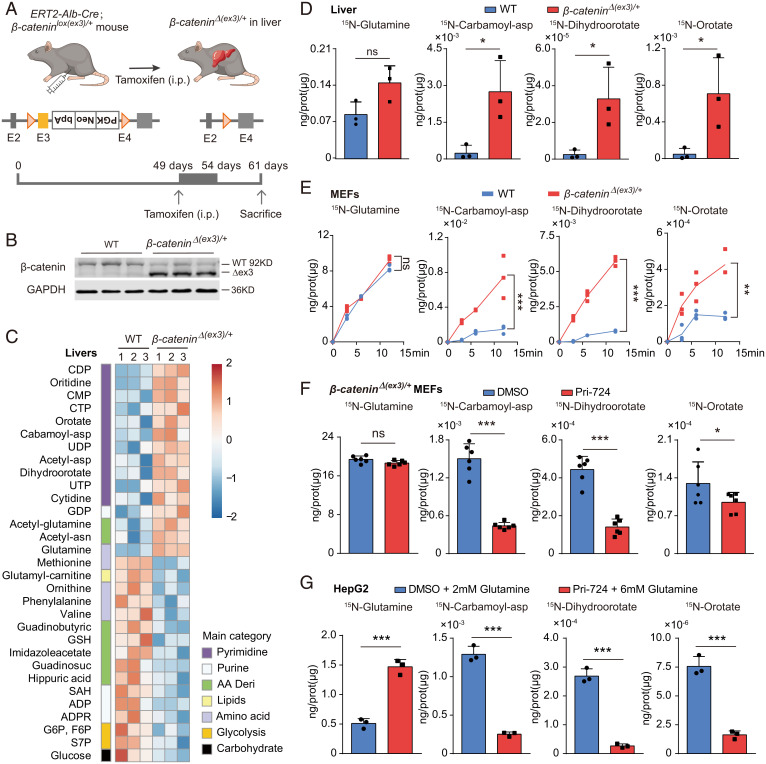
Oncogenic β-catenin stimulates de novo pyrimidine synthesis. (*A*) Generation of tamoxifen-induced hepatic β-catenin exon 3–deleted mice. Seven-week-old mice were injected with tamoxifen for 6 d and killed by day 12 for metabolic analysis. (*B*) Immunoblotting of WT and *β-catenin^Δ(ex3)/+^* livers. (*C*) Steady-state metabolite heatmaps of WT and *β-catenin^Δ(ex3)/+^* mouse livers. (*D*) ^15^N-labeled glutamine was used to trace pyrimidine synthesis in vivo. Abundance of ^15^N-labeled mouse liver metabolites 45 min after ^15^N-glutamine injection was analyzed. (*E*) The flux kinetics of ^15^N-labeled metabolites in MEFs at different time points after addition of ^15^N-glutamine. (*F* and *G*) Abundance of ^15^N-labeled metabolites. (*F*) *β-catenin^Δ(ex3)/+^* MEFs were treated with DMSO or Pri-724 (20 μM) for 24 h and a 12-min pulse labeling of ^15^N-glutamine (2 mM). (*G*) Pri-724–treated HepG2 cells were provided with excessive glutamine (6 mM) and DMSO-treated cells were provided with the standard dose of glutamine (2 mM). *P < 0.05, **P < 0.01, ***P < 0.001. Analysis was performed using *t* test. Data are shown as mean ± SD.

Liver is the major metabolic organ. Gain-of-function mutation of specific proto-oncogene often introduces metabolic vulnerabilities, which may be amenable to therapeutic intervention (16). To check oncogenic β-catenin–mediated cellular metabolic reprogramming without potential interference of nonactivated cells, we employed tamoxifen to induce expression of Alb-Cre recombinases to globally delete exon 3 of β-catenin in hepatocytes of *ERT2-Alb-Cre; β-catenin^lox(ex3)^*^/+^ mice (Fig. 3 *A* and *B* and *SI Appendix*, Fig. S5*A*). Untargeted metabolomic profiling was performed on both wildtype and *β-catenin^Δ(ex3)/+^* livers. Of 201 metabolites identified by liquid-chromatography (LC) mass spectrometry (MS) (Q-Exactive-plus), the abundances of 30 metabolites were different (*P* < 0.05) between wild-type and *β-catenin^Δ(ex3)/+^* livers (Fig. 3*C*). Because MEFs are widely used cell models for study of cell metabolism and signaling transduction (21, 22), we also carried out untargeted metabolomics for wild-type and β-catenin exon 3–deleted MEFs (*SI Appendix*, Fig. S5 *C* and *D*). Comparison of the metabolites enriched in *β-catenin^Δ(ex3)/+^* mouse livers and MEFs identified three most significantly elevated metabolites: carbamoyl-asp, dihydroorotate, and orotate, which are starting molecules for de novo pyrimidine biosynthesis pathway (Fig. 3*C* and *SI Appendix*, Fig. S5 *B*, *D*, and *E*).

De novo pyrimidine biosynthesis is a synthetic pathway that fuses nitrogen/carbon from glutamine, bicarbonate (HCO_3_^–^), and aspartate with ribose-phosphate to form heterocyclic nucleotides (*SI Appendix*, Fig. S5*F*). To elucidate active β-catenin–mediated regulation of intermediates’ biosynthesis, we traced the metabolic flux in vivo by injecting WT and hepatic *β-catenin^Δ(ex3)/+^* mice with ^15^N-amide glutamine, which is incorporated into the pyrimidine ring. Incorporations of ^15^N into multiple intermediates of pyrimidine synthesis were enhanced in β-catenin mutant livers over their wild-type counterparts (Fig. 3*D*). Consistently, the dynamic relative flux with ^15^N-labeled carbamoyl-asp, dihydroorotate, and orotate was dramatically increased in *β-catenin^Δ(ex3)/+^* MEFs (Fig. 3*E*) and the enhancement was compromised by Pri-724 treatment (Fig. 3*F*). Neither glutamine uptake nor expression of glutamine transporters was obviously altered by β-catenin in livers and MEFs (*SI Appendix*, Fig. S5 *A* and *C*), suggesting that β-catenin–regulated flux is not due to the change of glutamine availability. Furthermore, Pri-724 blocked pyrimidine synthesis in *CTNNB1* exon 3–deleted human HB cells (HepG2) and mouse HCC cells (Hepa 1-6) as well as human HCC cells (M97h and Huh7) (*SI Appendix*, Fig. S6).

It was reported that β-catenin transcriptionally activated GS (glutamine synthetase) to produce glutamine, which in turn activated mTOR in liver (23). S6K (P70S6K), the downstream effector of mTOR, potentiates pyrimidine synthesis (21). Therefore, we predicted that β-catenin stimulated pyrimidine synthesis through the potential GS-S6K connection. However, MEFs had no glutamate-derived glutamine and therefore no GS activity (*SI Appendix*, Fig. S5*G*), indicating that β-catenin might promote pyrimidine synthesis without GS involvement. Of note, the regulation of β-catenin on pyrimidine synthesis still existed after S6K depletion (*SI Appendix*, Fig. S6*E*). Moreover, the relative flux of pyrimidine synthesis was largely reduced by Pri-724 even in the presence of excessive glutamine (Fig. 3*G*). Therefore, oncogenic β-catenin stimulates pyrimidine synthesis without participation of the GS-S6K axis.

### β-Catenin Potentiates CAD Phosphorylation and Transactivates AKT2 Expression.

**Fig. 4.**
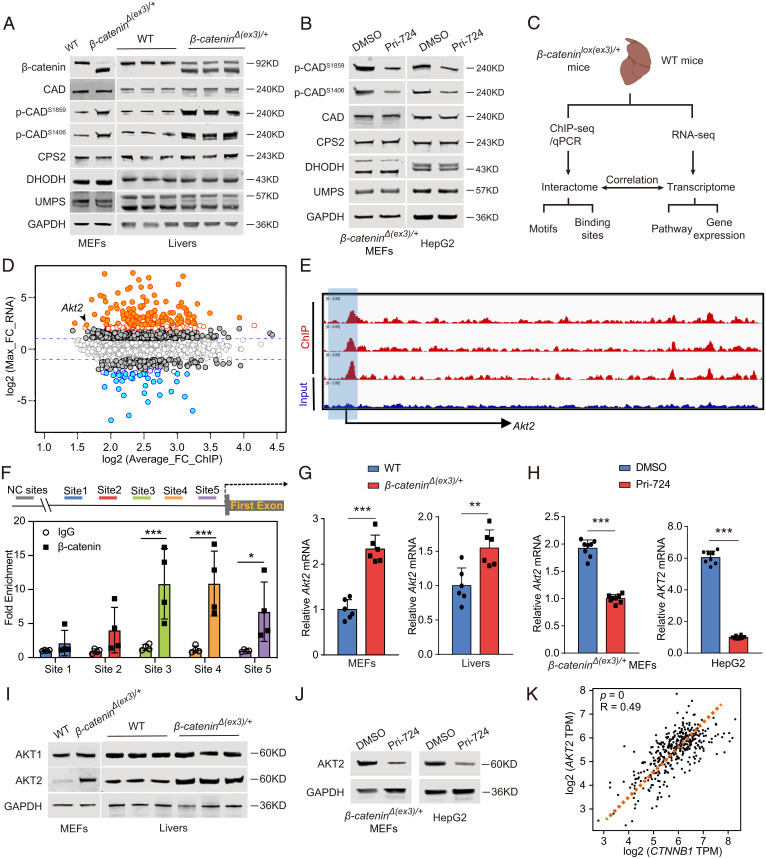
Oncogenic β-catenin potentiates CAD phosphorylation and promotes *Akt2* transcription. (*A*) Immunoblots of MEFs (*Left*) and livers (*Right*). (*B*) Immunoblots of *β-catenin^Δ(ex3)/+^* MEFs and HepG2 treated with DMSO or Pri-724. (*C*) Flowchart of RNA-seq and ChIP-seq of liver tissues. (*D*) Unbiased transcriptome analysis and interactome analysis by RNA-seq and ChIP-seq. (*E*) β-Catenin ChIP-seq profiles at *Akt2* loci. (*F*) β-Catenin ChIP-qPCR on *Akt2* promoter using various primers around peak sites from β-catenin ChIP-seq signals (*n* = 4 per group). Data are expressed as percent input retrieved, normalized to an upstream control site (NC site). (*G* and *I*) *Akt2* mRNA levels (*G*) and protein levels (*I*) in MEFs or mouse liver tissues. (*H* and *J*) *β-Catenin^Δ(ex3)/+^* MEFs or HepG2 were treated with DMSO or Pri-724, and qRT-PCR (*H*) or immunoblotting (*J*) was performed. (*K*) Correlation between *CTNNB1* and *AKT2* expression in human HCC. The original data were from TCGA database. *P < 0.05, **P < 0.01, ***P < 0.001. Analysis was performed using *t* test. Data are shown as mean ± SD.

To identify the mechanism by which oncogenic β-catenin stimulates pyrimidine synthesis, we checked the state of CAD, DHODH (dihydroorotate dehydrogenase), and UMPS (uridine monophosphate synthetase), the critical enzymes in this pathway. However, the abundances of their transcripts and proteins were not altered by β-catenin (*SI Appendix*, Fig. S7 *A* and *B* and Fig. 4 *A* and *B*). Carbamoyl-asp and dihydroorotate are catalyzed by CAD, whose activity strongly depends on the phosphorylation levels of its distinct sites (24). Therefore, we compared phosphoproteome between wild-type and *β-catenin* mutant livers as well as wild-type and *β-catenin* mutant MEFs to identify potential β-catenin–regulated phosphorylation sites of CAD. Among the critical phosphorylation sites for CAD enzymatic activity, S1859 and S1406 were highly phosphorylated in β-catenin mutant livers and MEFs (*SI Appendix*, Fig. S7 *C* and *D* and Table S3). Furthermore, hyperphosphorylation of S1859 and S1406 was abolished by Pri-724 (*SI Appendix*, Table S3). Immunoblotting demonstrated β-catenin stimulation of S1859 and S1406 phosphorylation on CAD in livers, MEFs, HepG2, Hepa 1-6, M97h and Huh7 cells (Fig. 4 *A* and *B* and *SI Appendix*, Fig. S7*E*). Of note, Pri-724 abolished phosphorylation of S1859 and S1406 on CAD even in the absence of S6K (*SI Appendix*, Fig. S7 *F* and *G*). Taken into consideration of S6K phosphorylation of S1859 but not S1406 on CAD (21), our data suggest the existence of other kinases responsible for β-catenin–promoted CAD phosphorylation.

Given that β-catenin is a transcriptional cofactor other than a protein kinase, we speculated that β-catenin promotes CAD phosphorylation by enhancing transcription of a potential CAD kinase(s). In quest of the putative kinase(s), we performed ChIP sequencing (ChIP-seq) and RNA-seq of mouse livers (Fig. 4*C*). β-Catenin possessed high specific chromatin association and potential transcriptional regulatory activity (*SI Appendix*, Fig. S8 *A–D*). Consistent with a previous study (25), β-catenin–binding peaks were enriched in *Glul* and *Axin2* genomic loci (*SI Appendix*, Fig. S8*E*). RNA-seq analysis was validated by the presence of β-catenin classical target genes, including *Glul*, *Oat*, and *Axin2* among the differentially expressed genes (*SI Appendix*, Fig. S8 *F* and *G*). Because *Akt2* presented in both the β-catenin up-regulated gene group (RNA-seq) and β-catenin–binding peak-enriched group (ChIP-seq) (Fig. 4*D* and *SI Appendix*, Fig. S8*G*), AKT2 was identified as the sole β-catenin transcriptionally up-regulated kinase. Notably, the S1859 region of CAD is predicted to be recognized by basophilic kinases such as AKT (26). Unbiased peak calling revealed that β-catenin bound to the *Akt2* promoter region (Fig. 4*E*), which was validated by ChIP-qPCR for several sites in the promoter region of *Akt2* (Fig. 4*F*). AKT2 expressed higher in β-catenin–activated MEFs and livers than in wild-type counterparts (Fig. 4 *G* and *I*). Furthermore, Pri-724 reduced AKT2 expression in β-catenin–mutated MEFs, HepG2, and Hepa 1-6 cells as well as in human HCC cells (M97h and Huh7) (Fig. 4 *H* and *J* and *SI Appendix*, Fig. S7*E*). Moreover, *AKT2* expression is positively corelated with *CTNNB1* expression in human liver cancer samples in The Cancer Genome Atlas (TCGA) (Fig. 4*K*). As all these findings indicate that β-catenin transcriptionally activates *AKT2* expression, we therefore speculated that AKT2 was the candidate kinase for CAD.

### AKT2 Is a Kinase of CAD.

**Fig. 5.**
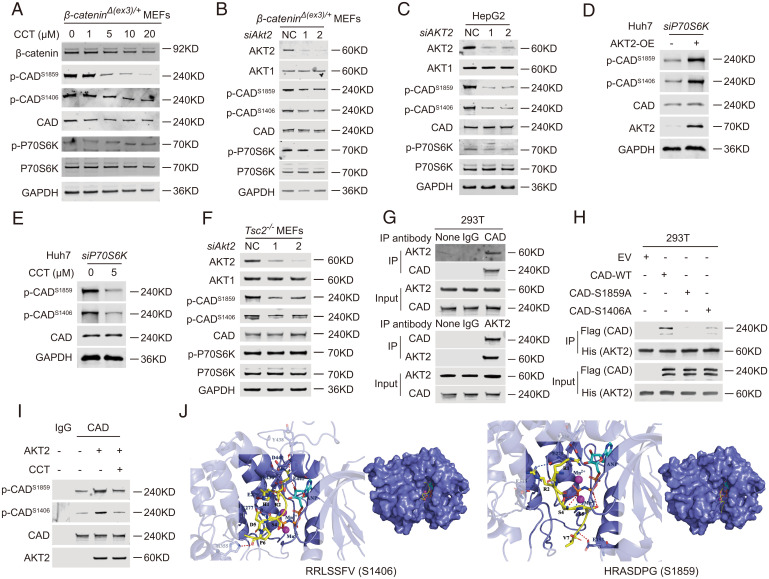
AKT2 is a kinase for CAD. (*A–I*) Immunoblotting. (*A*) *β-Catenin^Δ(ex3)/+^* MEFs were treated with DMSO or CCT for 6 h. *β-Catenin^Δ(ex3)/+^* MEFs (*B*) or HepG2 cells (*C*) were transfected with scramble or *AKT2* siRNAs. (*D*) After *S6K* siRNAs transfection, Huh7 cells were treated with DMSO or CCT (5 μM) for 6 h. (*E*) Control or AKT2-OE Huh7 cells were transfected with *S6K* siRNAs. (*F*) *Tsc2^−/−^* MEFs were transfected with scramble or *Akt2*-targeted siRNAs. (*G*) Coimmunoprecipitation (Co-IP) assays performed in 293 cells. (*H*) The 293 cells were cotransfected with vector (EV) or wild-type (WT), S1859A, or S1406A of *FLAG-HA-CAD* along with *HIS-HA-AKT2* vector for 48 h. Anti-HIS antibody-protein A/G magnetic beads were used for Co-IP. (*I*) In vitro kinase assays were performed with FLAG-HA-CAD proteins and AKT2 proteins with or without 1 μM CCT. (*J*) Docking analysis of AKT2 and RRLSSFV (containing S1406) or HRASDPG (containing S1859) of CAD protein. RRLSSFV and HRASDPG are in yellow. AKT2 is in slate. Mn^2+^ are shown as magenta sphere. ANP is in cyan. Red dashes represent hydrogen bond interaction, blue dashes represent salt bridge, and marine dashes represent metal contacts.

To examine whether AKT2 phosphorylates CAD, we first treated *β-catenin^Δ(ex3)/+^* MEFs with AKT2 kinase inhibitor, CCT128930 (CCT), for 6 h and then checked the status of CAD. We found that CCT reduced phosphorylation of S1859 and S1406 on CAD in a dose-dependent way (Fig. 5*A*). Consistently, AKT2 knockdown attenuated the phosphorylation of these two sites (Fig. 5*B*). Interference of AKT2 also reduced CAD phosphorylation in HepG2, M97h, and Huh7 (Fig. 5*C* and *SI Appendix*, Fig. S9 *A* and *B*). Because CAD is a substrate of S6K (a downstream effector of the AKT-mTOR signaling cascade), we examined potential S6K involvement in AKT2 stimulation of CAD phosphorylation. However, the regulation of AKT2 on CAD phosphorylation still presented when S6K was depleted by siRNA (Fig. 5 *D* and *E*). Furthermore, knocking down AKT2 also abolished phosphorylation of S1859 and S1406 on CAD in *Tsc2^−/−^* MEFs where the potential connection between AKT2 and S6K was disrupted (Fig. 5*F*). Moreover, low-dose treatment of CCT for 6 h abolished CAD phosphorylation without obviously affecting S6K phosphorylation in different cell types (Fig. 5*A* and *SI Appendix*, Fig. S9*A*). Taken together, AKT2 fosters CAD phosphorylation without involvement of S6K.

Next, we checked whether AKT2 directly phosphorylates CAD by biochemical assays. Co-IP assay revealed the interaction of endogenous CAD and AKT2 in various cell lines (Fig. 5*G* and *SI Appendix*, Fig. S9 *C–E*). AKT2 coimmunoprecipitated with exogenous wild-type, but not S1859A- or S1406A-mutated CAD (Fig. 5*H*). In vitro kinase assay showed that AKT2 phosphorylated S1859 and S1406 on CAD, which was abolished by CCT (Fig. 5*I*). Last, molecular docking analysis suggested that AKT2 interacted with S1859 containing peptide residues RRLSSFV and S1406 containing peptide residues HRASDPG in CAD protein (Fig. 5*J* and *SI Appendix*, Tables S4–S6). AKT2 is thus a kinase for CAD.

### AKT2 Stimulates De Novo Pyrimidine Synthesis.

**Fig. 6.**
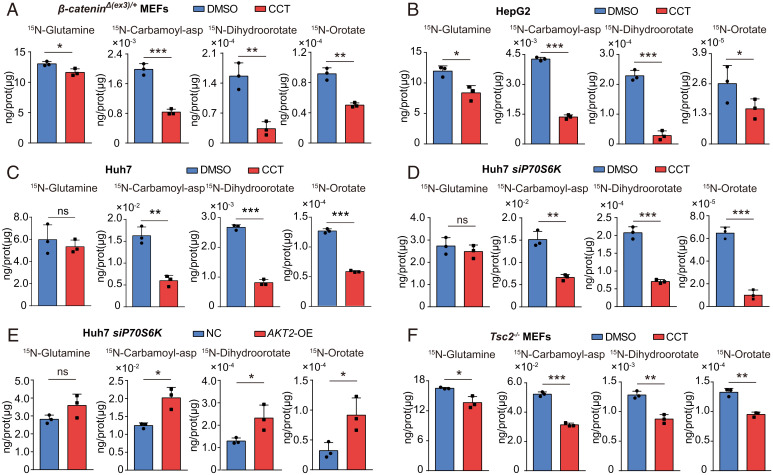
AKT2 stimulates de novo pyrimidine synthesis in various cell settings. Abundance of ^15^N-labeled metabolites. *β-Catenin^Δ(ex3)/+^* MEFs (*A*), HepG2 cells (*B*), Huh7 cells (*C*), and *Tsc2^−/−^* MEFs (*F*) were first treated with DMSO or CCT (5 μM) for 6 h and then pulse labeled with ^15^N-glutamine for 12 min. (*D*) At 48 h after *S6K* siRNAs transfection, Huh7 cells were first treated with DMSO or CCT (5 μM) for 6 h and then pulse labeled with ^15^N-glutamine for 12 min. (*E*) At 48 h after *S6K* siRNAs transfection, control or AKT2-OE Huh7 cells were pulse labeled with ^15^N-glutamine for 12 min. *P < 0.05, **P < 0.01, ***P < 0.001. Analysis was performed using *t* test. Data are shown as mean ± SD.

Since AKT2 phosphorylates CAD, which is a critical enzyme in stimulation of pyrimidine synthesis, we checked the role of AKT2 in pyrimidine synthesis. Suppression of AKT2 with CCT or *siAKT2* blocked the production of carbamoyl-asp, dihydroorotate, and orotate in β-catenin exon 3–deleted *β-catenin^Δ(ex3)/+^* MEFs (Fig. 6*A* and *SI Appendix*, Fig. S10*A*), HepG2 (Fig. 6*B*) and Hepa 1-6 cells (*SI Appendix*, Fig. S10*B*), as well as in Huh7 (Fig. 6*C*) and M97h cells (*SI Appendix*, Fig. S10*C*). Furthermore, AKT2 promoted pyrimidine synthesis, even when S6K was depleted (Fig. 6 *D* and *E*). In addition, interference of AKT2 abolished pyrimidine synthesis in *Tsc2^−/−^* MEFs, where the potential connection between AKT2 and S6K was interrupted (Fig. 6*F* and *SI Appendix*, Fig. S10*D*). To exclude the potential influence of moderate reduction of glutamine uptake on CCT-mediated inhibition of pyrimidine synthesis in some cell types (Fig. 6 *A*, *B*, and *F* and *SI Appendix*, Fig. S10 *B* and *C*), we provided CCT-treated cells with excessive glutamine (6 mM) and vehicle-treated cells with a standard dose of glutamine (2 mM). The relative flux of pyrimidine synthesis was largely reduced by CCT even in the presence of abundant glutamine (*SI Appendix*, Fig. S11). Taken together, AKT2 promotes de novo pyrimidine synthesis in various cell settings.

### Suppression of AKT2-Driven Pyrimidine Synthesis Abrogates Oncogenic β-Catenin–Mediated Cell Proliferation and Tumorigenesis.

**Fig. 7.**
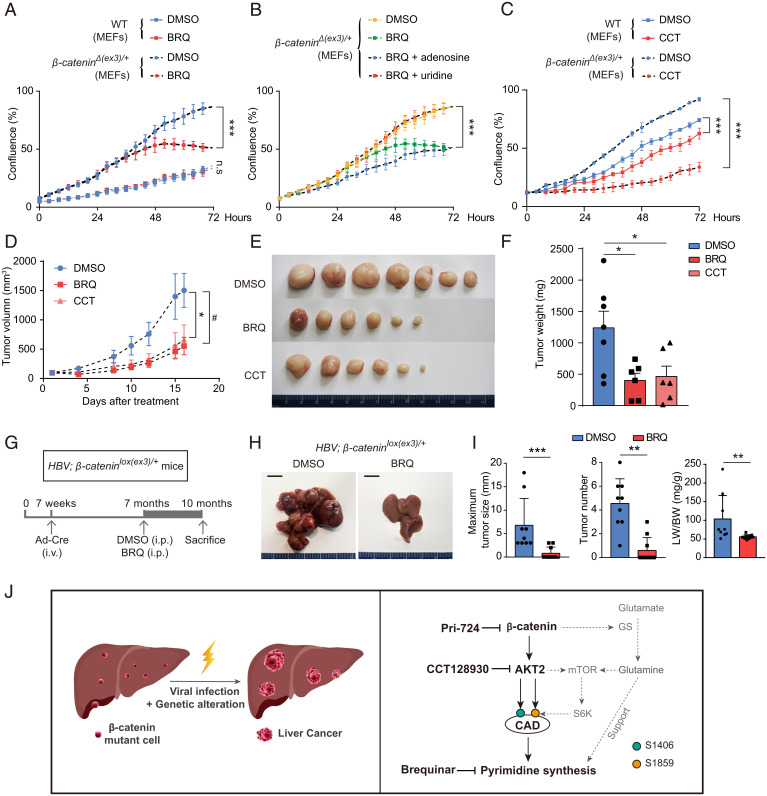
Suppression of AKT2-activated pyrimidine synthesis abrogates oncogenic β-catenin–mediated cell proliferation and tumorigenesis. (*A–C*) Cell proliferations of wild-type and *β-catenin^Δ(ex3)/+^* MEFs were measured with Incucyte assay. (*D–F*) Nude mice with subcutaneously inoculated *β-catenin^Δ(ex3)/+^* MEFs were treated with DMSO, BRQ (25 mg/kg, three times/wk), or CCT (25 mg/kg, five times/wk). (*D*) Tumor growth was plotted as the mean change in tumor volume. Tumor graphs (*E*) and tumor weights (*F*) at the end of treatment. (*G*) The 7-wk-old *HBV; β-catenin^lox(ex3)/+^* mice were injected via tail vein with *Cre*-adenoviruses. DMSO or BRQ treatment was started when mice were 7-mo-old and killed 3 mo later. (*H*) Representative livers from 10-mo-old *HBV; β-catenin^lox(ex3)/+^* mice. (*I*) The maximum liver tumor size (*Left*), tumor number (*Middle*), and ratio of liver weight to body weight (*Right*) were analyzed. (*J*) Schematic illustration of oncogenic β-catenin stimulating pyrimidine synthesis in promotion of hepatocarcinogenesis. *, ^#^*P* < 0.05. Analysis was performed using *t* test (*A–D*, and *I*) and one-way ANOVA (*F*). Data are shown as mean ± SD.

To explore therapeutic relevance of AKT2 stimulation of pyrimidine synthesis, we examined the efficacy of AKT2 or pyrimidine synthesis inhibitor in suppression of cell proliferation and tumorigenesis driven by oncogenic β-catenin. Compared to wild-type MEFs, *β-catenin^Δ(ex3)/+^* MEFs were more sensitive to proliferation inhibition with Brequinar (BRQ) (Fig. 7*A*), a DHODH inhibitor, which depletes pyrimidine nucleotides. BRQ-mediated repression of proliferation was reversed by uridine (a pyrimidine recovery metabolite) but not adenosine (a purine recovery metabolite) (Fig. 7*B*). *β-Catenin^Δ(ex3)/+^* MEFs were more sensitive than wild-type MEFs to CCT treatment (Fig. 7*C*). In addition, either BRQ or CCT suppressed tumorigenicity of *β-catenin^Δ(ex3)/+^* MEFs without affecting body weight in nude mice (Fig. 7 *D–F* and *SI Appendix*, Fig. S12). With moderate influence on migration and invasion, β-catenin, AKT2, or pyrimidine synthesis inhibitor dramatically repressed proliferation and promoted apoptosis of HepG2, Huh7, and M97h cells (*SI Appendix*, Figs. S13 and S14). Moreover, BRQ blocked liver tumor initiation and advancement of *HBV; β-catenin^lox(ex3)/+^* mice injected with *Cre*-adenoviruses (Fig. 7 *G–I*) without significant influence on body weight and organs except enlarged spleen (*SI Appendix*, Fig. S15). Therefore, targeting AKT2-potentiated pyrimidine synthesis may be a promising therapeutic strategy for *β-catenin* mutant liver cancer.

## Discussion

*CTNNB1* is the most frequently mutated proto-oncogene in liver cancer (4). We demonstrated that hepatic β-catenin active mutation caused mouse liver cancer and accelerated transgenic *HBV* or *Tp53* deletion-induced mouse liver cancer development. In addition, we discovered a β-catenin/AKT2/CAD signaling axis in enhancement of de novo pyrimidine synthesis, which was critical for hepatocarcinogenesis. The causality of the *CTNNB1* mutation and hepatocarcinogenesis thus confers AKT2/CAD signaling cascade-mediated pyrimidine synthesis a druggable vulnerability for *CTNNB1* mutant cancer.

Being a benign liver neoplasm with risk of malignant transformation, HCA has more than 10% frequency of *CTNNB1* mutation (27). With over half of the patients carrying *CTNNB1* mutations, HB is the most common malignant liver tumor in childhood (5). More importantly, *CTNNB1* gain-of-function mutations are detected in more than 20% liver cancer. Standard whole exome sequencing analysis easily detects single nucleotide variants and short indels but not intermediate-size indels (≥50 bp) (28). Small variants are readily detected but not the frequent intermediate-size DNA deletions around exon 3 regions of *CTNNB1*. Several studies have pointed out that intragenic deletions of *CTNNB1* cannot be detected by next generation sequencing analysis pipelines (28, 29). Therefore, the actual frequency of *CTNNB1* mutations is likely underestimated in HCC. Even though *CTNNB1* is the most common mutated proto-oncogene, the significance of hyperactive β-catenin in liver cancer was not well documented (7). However, the *CTNNB1* mutation is sufficient to cause liver cancer in zebrafish (30). Therefore, we were compelled to investigate the causative relationship between *Ctnnb1* aberration and liver tumors in mice. While we and others did not detect liver tumors up to 6 mo after hepatic *Ctnnb1* exon 3 deletion (9), we observed spontaneous tumor formation 11.5 mo after β-catenin activation in mouse liver in this study, similar to a very recent report (31). Therefore, the reason that previous study did not observe liver tumors caused by *Ctnnb1* mutations in mice is due to insufficient observation time. Because HCA usually occurs in people with elevated estrogen such as women taking estrogen-containing oral contraceptives, mouse livers with activated β-catenin alone did not authentically mimic HCA in this study. Moreover, early lethality caused by albumin promoter-driven *Cre-*recombinase–induced global hepatic *Ctnnb1* exon 3 deletion and consequent β-catenin activation preclude us from faithfully recapitulating HB, a mosaic β-catenin mutation-associated liver cancer usually seen in infants and young children under age 3, in the mice we generated. By simulating somatic mutation of *CTNNB1* in patients, we nevertheless demonstrate that activated β-catenin causes both benign and malignant tumors in mice.

Because β-catenin–activated mouse livers developed HCC over a long period of time, we speculated that additional genetic alterations or insults were required for liver malignant transformation. *TP53* is the most mutated tumor suppressor in overall cancer. It is also the highest mutated gene (29%) in liver cancer (4). Hepatic *Tp53*-deficient mice develop liver tumors when they are 14 to 20 mo old (32). As half of *CTNNB1* mutant liver cancer harbors *TP53* mutation (*SI Appendix*, Table S2) (15, 18–20), we simultaneously activated β-catenin by deleting its exon 3 and inactivated TP53 by knocking it out in mouse liver. These mice grew HCC much earlier than either β-catenin mutant mice or *Tp53* knockout mice. In addition, *Ctnnb1* mutation also potentiated tumorigenic potential of *Tp53* null MEFs. Therefore, loss of TP53 synergizes with gain of function of β-catenin in potentiation of liver cancer progression. Further studies are needed to elucidate the mechanism of complicity between these two distinct signaling pathways.

HBV infection is the major risk factor for liver cancer (11). Although China has more than 93 million HBV carriers, only a small fraction of HBV-infected people develop liver cancer over a long period of latency. In addition, *HBV* transgenic mice grow liver tumors more than 12 mo after birth (33). Therefore, progression from hepatitis B infection to liver cancer requires other carcinogenic events. Previous studies observed mutant *CTNNB1* in up to 19% of HBV-related HCC (15). Because mutant β-catenin strongly drove hepatocarcinogenesis of *HBV* transgenic mice in our study, we suggest that active β-catenin contributes to the pathological process of HBV-associated HCC. Blockade of β-catenin with Pri-724 abrogated liver tumorigenesis in mice with concurrent transgenic *HBV* and mutated *Ctnnb1*. By mimicking clinical settings with HBV infection, or *TP53* inactive mutation, along with *CTNNB1* active mutation in liver, we simulated human multiple-step pathological processes of hepatocarcinogenesis in mice.

One of the hallmarks of cancer is metabolic reprogramming, which provides macromolecules to meet the unchecked proliferation of tumor cells. We dissected the metabolic alterations of oncogenic β-catenin in MEFs and mouse livers. The most dramatically distorted metabolic pathway identified is the de novo pyrimidine synthesis pathway. Mechanistically, transcriptionally activated AKT2 by β-catenin directly phosphorylates S1859 and S1406 on CAD to promote pyrimidine synthesis. Targeting the β-catenin/AKT2/CAD axis repressed cell proliferation and tumor development driven by oncogenic β-catenin. Since β-catenin transcriptionally regulates many signaling pathways, this transcription coactivator has widely biological and pathological functions (7). The β-catenin/AKT2/p-CAD axis we discovered and presented here is one of the critical signaling cascades underlying β-catenin activation-mediated hepatocarcinogenesis. Pyrimidine synthesis has been recently discovered as a metabolic vulnerability and promising therapy target in KRAS/LKB1 mutant non-small-cell lung cancer and PTEN mutant cancer (34, 35). Our findings illustrate the importance of pyrimidine synthesis in β-catenin mutant cancer. Recently, combining anti–PD-L1 antibody atezolizumab with anti–VEGF-A antibody bevacizumab achieved promising efficacy for advanced HCC patients (36, 37). Aberrant β-catenin activation promotes immune escape and resistance to immunotherapy in HCC (38–40). Increasing evidence indicates that targeting nucleotide metabolism, including pyrimidine synthesis, can enhance the antitumor response of immunotherapy (41–44). Therefore, targeting AKT2/pyrimidine synthesis is warranted to overcome β-catenin activation–associated resistance of immunotherapy for HCC.

It was reported that β-catenin transcriptionally activated GS to produce glutamine, which in turn activated mTOR in liver (23). S6K (P70S6K), the downstream effector of mTOR, potentiates pyrimidine synthesis (21). An outstanding question is whether the β-catenin/AKT2 axis relies on the GS-S6K signaling cascade to promote pyrimidine synthesis. We demonstrated that regulation of pyrimidine synthesis by the β-catenin/AKT2 cascade still exists in MEFs, which lacks GS enzymatic activity, in S6K-depleted liver cancer cells, and in *Tsc2* null cells where potential activation of β-catenin/AKT2 on S6K is disconnected. While S6K only phosphorylates CAD on S1859 (21), we found that β-catenin–transactivated AKT2 phosphorylates both S1406 and S1859 on CAD. Therefore, the β-catenin/AKT2 axis can directly stimulates pyrimidine synthesis without the involvement of S6K. We suggest that oncogenic β-catenin stimulates pyrimidine synthesis possibly through different routes: 1) β-Catenin transcriptionally activates AKT2 and the enhanced AKT2 then promotes pyrimidine synthesis either by directly phosphorylating CAD or by activating mTOR-S6K. 2) In hepatocytes, where GS has the normal activity of glutamine synthetase, β-catenin also promotes GS expression to maintain a high level of glutamine, which can either stimulate mTOR-S6K or serve as substrate to support pyrimidine synthesis (Fig. 7*J*). The β-catenin/AKT2/CAD pathway we discovered is thus an alternative regulatory mechanism to the known regulation of the mTOR-S6K cascade on de novo pyrimidine synthesis.

In summary, β-catenin is a major oncogenic driver for the development of hepatic tumors, including HCA, HB, and HCC. AKT2-mediated CAD phosphorylation is required for β-catenin–induced pyrimidine synthesis and hepatocarcinogenesis. Pharmacological inhibition of β-catenin, AKT2, and/or pyrimidine synthesis abolishes β-catenin mutant liver cancer. Hence, these readily translatable strategies warrant prompt clinical trials.

## Materials and Methods

### Mice.

*β-Catenin^lox(ex3)^*^/+^ mice were kindly provided by Haibin Wang, Xiamen University, Xiamen, Fujian, China (8). *HBV* transgenic mice (33) were purchased from the Department of Laboratory Animal Science, Peking University Health Science Center. *Tp53^flox/flox^* mice (*Tp53^l/l^*) (32) were purchased from Model Animal Research Center, Nanjing University. *Alb-Cre* mice (stock no. 003574) was from The Jackson Laboratory. *ERT2-Alb-Cre* mice were generated by Biocytogen. To delete exon 3 of *Ctnnb1* in mouse liver 7 wk after birth, 6 × 10^8^ pfu *Cre-*adenovirouses (#1700, Vector Biolabs) were intravenously injected into *β-catenin^lox(ex3)^*^/+^ mice as previously reported (9). The 5-mo-old *HBV; β-catenin^lox(ex3)^*^/+^ mice were intraperitoneally injected with Pri-724 (5 mg/kg) every other week for 3 mo. The 7-mo-old *HBV; β-catenin^lox(ex3)^*^/+^ mice were intraperitoneally injected with BRQ (25 mg/kg, 3 times/wk) for 3 mo. *ERT2-Alb-Cre* mice were crossed with *β-catenin^lox(ex3)^*^/+^ mice to generate inducible *ERT2-Alb-Cre; β-catenin^lox(ex3)^*^/+^ mice. To delete hepatic exon 3 of *Ctnnb1*, these mice were injected intraperitoneally with 30 mg/kg tamoxifen for 6 consecutive days and killed by day 12 for analyses. The experimental protocols of all animal studies were approved by the Animal Center of the Institute of Basic Medical Sciences, Chinese Academy of Medical Sciences and Peking Union Medical College (ACUC-A02-2014-003). Experimental procedures were compliant with the regulation of Beijing Administration Office of Laboratory Animals.

### Metabolomic Extraction of Mouse Liver.

Livers were excised and immediately processed for metabolomics extraction. Equal amounts of liver tissue were lysed in 250 μL of extraction solution (ES, 80% methanol and 20% water) per 10 mg of tissue in Precellysis vials following the manufacturer’s instruction. The suspension was immediately centrifuged at 16,000 × *g* for 15 min at 4 °C. The supernatant was then subjected to LC-MS metabolomic analysis.

### Metabolomic Extraction of Cells.

Cells were plated onto six-well plates (5 × 10^5^/well) and cultured in Dulbecco's modified eagle medium for 24 h. The cell number was counted in a parallel control dish. For intracellular metabolomic analysis, cells were quickly washed three times with Phosphate buffer saline and were then lysed in 1 mL precooled extraction solution per 1 × 10^6^ cells. The cell lysates were vortexed for 5 min at 4 °C and immediately centrifuged at 16,000 × *g* for 15 min at 0 °C. The supernatants were collected and analyzed by LC-MS metabolomic analysis. Total protein contents of cell pallets were also determined.

### Statistical Analysis.

Real-time mRNA expression, tumor-free, mouse-survival, tumor-burden, and metabolites data were analyzed with GraphPad Prism 9.0. The data presented were mean ± SD. Survival or tumor-free data of mice was compared among various groups using a log-rank (Mantel–Cox) test. Statistical significance between two groups was subjected to Student’s *t* test. One-way ANOVA was used to determine the difference in more than two groups. *P* < 0.05 was considered statistically significant.

## Supplementary Material

Supplementary File

## Data Availability

ChIP-seq and RNA-seq data have been deposited in Gene Expression Omnibus (GEO), ChIP-seq: GSE165853 (45) and RNA-seq: GSE188957 (46). All other study data are included in the article and/or *SI Appendix*.
