# Supplementary material for: Oncogenic β-catenin stimulation of AKT2–CAD-mediated pyrimidine synthesis is targetable vulnerability in liver cancer
Source: Proc Natl Acad Sci U S A. 2022 Sep 19;119(39):e2202157119. doi: 10.1073/pnas.2202157119 (PMC9522414; doi:10.1073/pnas.2202157119)
Supplement: Supplementary File [file pnas.2202157119.sapp.pdf]

PNAS

Supplementary Information for
Oncogenic β-catenin stimulation of AKT2-CAD-mediated pyrimidine synthesis is targetable vulnerability in liver cancer

Authors: Fangming Liu,[1,#] Xiaochen Gai,[1,#] Yuting Wu,[1] Baohui Zhang,[2] Xiaoyu Wu,[3] Rongrong Cheng,[3] Bufu Tang,[4] Kezhuo Shang,[1] Na Zhao,[1] Weiwei Deng,[1] Jie Chen,[5] Zhengyi Zhang,[6,7] Song Gu,[8] Liang Zheng,[3,*] Hongbing Zhang[1,*]

[*]**Correspondence:** Hongbing Zhang, hbzhang@ibms.pumc.edu.cn. Tel: 01186-10-69156495.
Liang Zheng, zhengliang@scmc.com.cn. Tel: 01186-21-38087439

This PDF file includes:

Supplementary text
Figures S1 to S15
Tables S1 to S6
SI References

Supplementary Information Text

Materials and Methods

Histology and immunohistochemistry (IHC)

Tissues were first fixed with paraformaldehyde for subsequent analysis. H&E staining, reticular fiber staining and IHC were performed by Servicebio (Wuhan, Hubei, China). Sections were analyzed using a Pannoramic DESK microscope with Caseviewer (version C.V 2.3).

Antibodies, inhibitors, and other agents

Anti-β-catenin (#9587), anti-p-CAD Ser^{1859} (#7030), anti-AKT2 (#2964), anti-AKT1 (#2938), anti-P70S6K (#2708), anti-p-P70S6K Thr^{389} (#9205), anti-CAD (#93925), anti-SLC38A1 (#36057), anti-Vimentin (#5741), and secondary HRP-conjugated mouse anti-rabbit IgG (#5127) antibodies were purchased from Cell Signaling Technology (Danvers, MA, USA). Anti-GS (#610517) antibody was purchased from BD Biosciences (San Jose, CA, USA). Anti-OAT (#A6235), anti-UMPS (#A13251), anti-DHODH (#A6899), anti-SLC1A5 (#A6981), anti-LGR5 (#A10545), anti-AXIN2 (#A2513), anti-CYP2E1 (#A2160), anti-Cyclin D1 (#A19038) and anti-GAPDH (#AC002) antibodies were purchased from Abclonal Technology (Wuhan, Hubei, China). Anti-CPS2 (#376072) was purchased from Santa Cruz Biotechnology (Santa Cruz, CA, USA). Anti-SLC38A2 (PA5106786) was purchased from Invitrogen. Anti p-CAD Ser^{1406} was ordered from PTM BioLab (Hangzhou, Zhejiang, China). Secondary IRDye 680RD goat anti-rabbit (#68071) and IRDye 800 CW goat anti-mouse (#32210) antibodies were purchased from LI-COR Biosciences (Lincoln, NE, USA). Brequinar (BRQ; #96187-53-0) was purchased from TargetMol (Wellesley Hills, MA, USA). CCT-128930 (CCT; #2635) and Pri-724 (#8262) were purchased from Selleck Chemicals (Houston, TX, USA). Dulbecco's modified Eagle's medium (DMEM), Fetal bovine serum (FBS), Lipofectamine 2000, Trizol, 4–12% Bis–Tris NuPAGE gels, Penicillin-Streptomycin and Trypsin-EDTA were purchased from Invitrogen (Carlsbad, CA, USA). Cell Cycle and Apoptosis Analysis Kit (No. 40301) and CCK-8 agent (40203ES60) were purchased from Yeasen Biotech (Shanghai, China)

siRNA transfection

The small interfering RNA oligonucleotides against mouse/human *AKT2* and *P70S6K* genes were purchased from Hippo Biotechnology (Hangzhou, Zhejiang, China). siRNA transfection was carried out using lipofectamine 2000 following the manufacturer's instructions. The miRNA sequences are listed below.
si-AKT2-1: 5′-UUGUACCCAAUGAAGGAGCCG-3′
si-AKT2-2: 5′-AUUGUGAUGGACUGGGCGGUA-3′
si-P70S6K-1: 5′-AUGGAACAUUGUGAGAAAUUUGA-3′
si-P70S6K-2: 5′- AACAUUGUGAGAAAUUUGAAAUC-3′
si-P70S6K-3: 5′- GGCUAUGGAAAGGUUUUUCAAGU-3′
Negative control: 5′-ACGUGACACGUUCGGAGAA-3′

Real-time quantitative reverse transcription PCR

Total RNA was isolated using Trizol reagent. cDNA synthesis was performed using ReverTra Ace qPCR RT Master Mix with gDNA Remover kit (Toyobo, Osaka, Japan) following manufacturer's instruction. RT-PCR was carried out using SYBR High-Sensitivity qPCR SuperMix (Absin Biosciences, Shanghai, China) on CFX96 Touch (BioRad, Hercules, CA, USA). Each mRNA expression was normalized to *Actin* mRNA expression. The primer sequences were as followed:

mouse *Umps*
forward: 5′-GTCACCGAGCTGTATGACGTG-3′
reverse: 5′-GGTAACGCTGTATAAGGAACTCC-3′
mouse *Dhodh*
forward: 5′-TCTTCACCTCTTACCTGACAGC-3′
reverse: 5′-CATGTTGGAGTCCTGAAACGTA-3′
mouse *Cad*
forward: 5′-CTGCCCGGATTGATTGATGTC-3′
reverse: 5′-GGTATTAGGCATAGCACAAACCA-3′
mouse *Akt2*
forward: 5′-ACGTGGTGAATACATCAAGACC-3′
reverse: 5′-GCTACAGAGAAATTGTTCAGGGG-3′
mouse *Actin*
forward: 5′-AGAGGGAAATCGTGCGTGAC-3′
reverse: 5′-CAATAGTGATGACCTGGCCGT-3′
human *AKT2*
forward: 5′- GGTGCAGAGATTGTCTCGGC-3′
reverse: 5′- GCCCGGCCATAGTCATTGTC-3′
human *ACTIN*
forward: 5′- GAGCTGCGTGTGGCTCCC -3′
reverse: 5′- CCAGAGGCGTACAGGGATAGCA -3′

Primary mouse embryonic fibroblasts

β-catenin$^{lox(ex3)/+}$ mice were crossed with wildtype mice to produce embryos which were then used to establish *WT* and *β-catenin*$^{lox(ex3)/+}$ mouse embryonic fibroblasts (MEFs), respectively. For deletion of exon 3 of *β-catenin*, MEFs were infected with adenovirus expressing Cre recombinase (#1700, Vector Biolabs).

Incucyte assay

Cells were seeded at a density of 4000 cells/well in 96-well plates. Cell proliferation was monitored using IncuCyte ZOOM live cell analysis system (Essen Bioscience, Ann Arbor, MI, USA).

Co-immunoprecipitation and immunoblotting

293 cells were grown in 15-cm dishes to 80-90% confluency and then lysed in NP-40 lysis buffer (Abclonal) supplemented with cocktail protease and phosphatase inhibitor (Roche, Basel, Switzerland) on ice for 10

min. After centrifugation at 14,000 g for 10min at 4°C, the collected supernatants were incubated with protein A/G beads for 10 min at 4°C to remove non-specific binding components. Total protein was used as input. For IPs, total protein lysate was rotated with anti-IgG control (10μg), anti-CAD and anti-AKT2 antibodies at 4°C overnight. Next, protein A/G beads (50μL per sample) were added and incubated for 30 min at room temperature. After washing with PBST [PBS and 0.5% Triton X-100 (pH7.4)] for 3 times, samples were resuspended in 40μL of SDS sample loading buffer, heated at 95°C for 5 min before loading for SDS-PAGE. Lysates were separated by NuPAGE 4 to 12% bis-tris gels and transferred to nitrocellulose membranes (Merck Millipore, Darmstadt, Germany). Membranes were blocked with blocking buffer (LI-COR Biosciences) for 1 hour, incubated with primary antibodies overnight at 4℃, washed for 10 min in tris-buffered saline (TBST) buffer [10mM tris-HCl (pH 8), 150mM NaCl, and 0.05% Tween 20] for 3 times and then incubated with secondary antibodies for 2 hours at room temperature. After washes 3 times in TBST, membranes were then developed using SuperSignal enhanced chemiluminescence (LI-COR Biosciences).

Subcutaneous tumor assay

MEFs (1×10^6 cells per mouse) were subcutaneously injected into the right flanks of BALB/c nude mice (female, 4-6 weeks old, 6–8 mice per group). Tumor volume was calculated according to formula: tumor volume =1/2 (length×width^2). Once the tumor volume developed to around 100 mm^3, the mice were randomly assigned for intraperitoneal treatment of vehicle, BRQ (25mg/kg) or CCT (25mg/kg) for 3 times per week. Tumor lengths and widths were measured every other day. When the maximum volume reached to 1,000mm^3, tumors were harvested, weighed, and photographed.

LC-MS metabolomic analysis

For LC separation, column A was Merck Zic-Hilic (150mm 4.6mm, internal diameter (i.d.) 5mm) (Darmstadt, Germany). Mobile phase A was 0.1% formic acid v/v in water. Mobile phase B was 0.1% formic acid v/v in acetonitrile. The flow rate was kept at 300μL/min and gradient was as follows: 0 min 80% of B, 12 min 50% of B, 26 min 50% of B, 28 min 20% of B, 36 min 20% of B, 37–45 min 80% of B. Column B was Merck Zic-pHilic (150mm 2.1mm i.d. 5mm). Mobile phase C was 20mM ammonium carbonate plus 0.1% ammonia hydroxide in water. Mobile phase D was acetonitrile. The flow rate was kept at 100μL/min and gradient as follow: 0 min 80% of D, 30 min 20% of D, 31 min 80% of D, 45 min 80% of D. The mass spectrometer (Thermo Exactive Orbitrap) was operated in a polarity switching mode. The data was analyzed by Thermo Compound Discoverer 3.1 and Tracefinder 4.0.

Phosphorylated proteomics

Phosphorylated proteomics of mouse livers and MEFs were carried out by PTM BioLab. The resulted MS/MS data were processed using Maxquant search engine (v.1.5.2.8). Tandem mass spectra were searched against Human uniprot database concatenated with reverse decoy database. Trypsin/P was specified as cleavage enzyme allowing up to 4 missing cleavages. The mass tolerance for precursor ions was set as 20 ppm in First search and 5 ppm in Main search, and the mass tolerance for fragment ions was set as 0.02 Da. Carbamidomethyl on Cys was specified as fixed modification and acetylation modification

and oxidation on Met were specified as variable modifications. FDR was adjusted to < 1% and minimum score for modified peptides was set > 40.

RNA-seq

WT and *β-catenin*$^{Δ(ex3)/+}$ mouse livers (3 per group) were used for RNA-seq. Total RNA was extracted using Trizol reagent (Thermo Fisher, 15596018) following the manufacturer's protocol. High-quality RNA samples with RIN number > 7.0 were used to construct sequencing library. RNA-seq was performed by Illumina Novaseq^{TM}6000 sequence platform (LC-Bio Technology, Hangzhou, Zhejiang, China). Genes differential expression analysis was performed by DESeq2 software between two different groups. The genes with the parameter of false discovery rate (FDR) below 0.05 and absolute fold change≥2 were considered differentially expressed genes. These differentially expressed genes were then subjected to enrichment analysis of GO functions and KEGG pathway.

ChIP-seq and ChIP-PCR

ChIP experiments were performed based on previously described protocol with minor revision to analyze the binding partners of β-catenin (1). Three *β-catenin*$^{Δ(ex3)/+}$ mouse livers were used for β-catenin ChIP-seq. Livers were sonicated using a M220 Focused-ultrasonicator (Covaris, Woburn, MA, USA). Chromatin was immunoprecipitated with 4μg antibodies against β-catenin (Carboxy-terminal Antigen, #9587S) overnight at 4°C. ChIP-Seq libraries were prepared using Kapa LTP Library Preparation Kit (Kapa Biosystems, Sigma-Aldrich). ChIP-Seq was performed on Illumina HiSeq3000. For the bioinformatic analysis of ChIP-Seq data, Bowtie2 was used for alignment. Enriched peaks were identified using MACS2 with FDR < 0.01 (2-4). ChIPseeker was used for peaks annotation (5). Peaks enrichment distribution was analyzed by Deeptools (6). For β-catenin ChIP-PCR, 4 replicates were performed. Briefly, 80-100mg of wild-type mouse livers were used as chromatin samples which were prepared using ChIP Chromatin Shearing Tissue Kit (Covaris). Sonication was performed using a M220 Focused-ultrasonicator (Covaris) according to the manufacturer’s protocol for 15 min, and chromatin was immunoprecipitated with 4μg antibodies against β-catenin overnight at 4°C. The ChIP samples were analyzed by real-time PCR using primers as followed. All values obtained were normalized to the primers of negative control region.

NC
forward: 5′-GTTTGAAGTTTGGGCGGTCC-3′
reverse: 5′-CTGAACCCCTTAGGGTGACG-3′
Site 1
forward: 5′-TGACCTCATGGGGTGAACTG-3′
reverse: 5′-CCACCTCAAGGTCTTTGCCT-3′
Site 2
forward: 5′-AGGGCGTTAGATTGTGAGCC-3′
reverse: 5′-AGGTTAAGAGCACCGACTGC-3′
Site 3
forward: 5′-CAGGTATGTACACCCGCTGA-3′

reverse: 5′-TATGCCAGCCTAGGCAAGTG-3′
Site 4
forward: 5′-CCCATGGCGCTGACTACAG-3′
reverse: 5′-AGTAGATGCAAGTGAGGAGCC-3′
Site 5
forward: 5′-GAACCGCTGGTCTAGGAACT-3′
reverse: 5′-TACTTCCCGGCAATCAAAGGG-3′

CCK8 assay

Cells (5000 in each well) were seeded in 96-well plates and were treated with DMSO, Pri-724 (20μM), CCT (5μM) or BRQ (5μM). Cell viability was measured 48 h later by Cell Counting Kit-8 (Selleck Biotechnology, TShanghai, China). The absorbance at 450 nm wavelength was determined.

Cell apoptosis assay

HepG2, M97h and Huh7 cells were treated with DMSO, Pri-724 (20μM), CCT (5μM) or BRQ (5μM) for 48 h. The floating apoptotic cells in supernatant were collected and the adherent cells were trypsinized. Apoptosis of total cells was determined by flow cytometry using Annexin V-FITC Apoptosis Detection Kit (Beyotime, Shanghai, China).

Cell migration assay

Cells were seeded into 6-well plates and were treated with DMSO, Pri-724 (20μM), CCT (5μM) or BRQ (5μM). Wound was generated by scratching across the cell monolayer with sterilized pipette tip and the cellular debris was washed away with PBS. Cell migration or protrusion from the wound boarder was measured and photographed under inverted microscope at 0 and 48 h.

Transwell assay

Cells (2 x 10^4 for M97h and Huh7 cells, 4 x 10^4 for HepG2 cells) were loaded in the upper chamber of Transwell plates (8 mm pore size; BD Biosciences, LA, USA) in DMEM without serum. And these cells were treated with DMSO, Pri-724 (20μM), CCT (5μM) or BRQ (5μM). The lower chamber was filled with 15% FBS/DMEM. After 24 h, cells passing through the membrane were stained with 1% trypan blue and counted using a microscope (7).

Figure S1

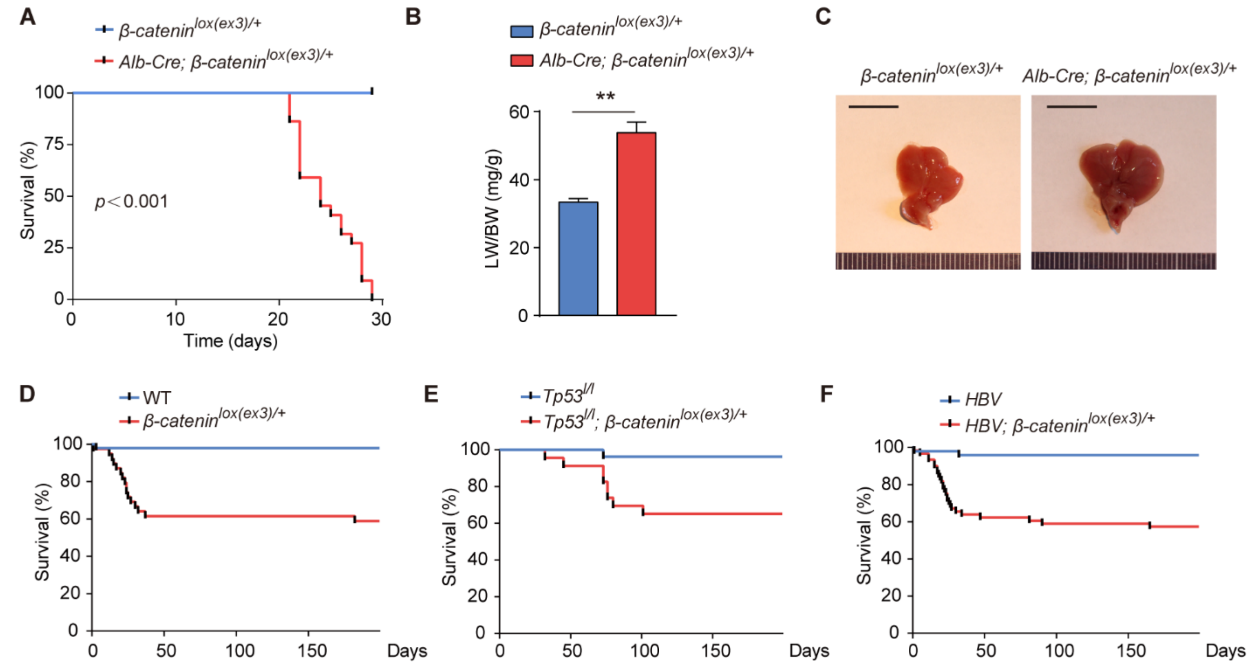

Fig. S1 Hepatic β-catenin activation leads to acute mouse death.

(A-C) Albumin-cre-mediated hepatic β-catenin exon 3 deletion. Survival *(A)*, ratio of liver weight to body weight (*B*), and representative liver pictures *(C)* were presented. *(D-E)* After *Cre*-adenoviruses tail vein injection, WT and *β-catenin*$^{lox(ex3)/+}$mice *(D)*, *HBV* and *HBV; β-catenin*$^{lox(ex3)/+}$ mice *(E)*, *Tp53*$^{l/l}$ and *Tp53*$^{l/l}$*; β-catenin*$^{lox(ex3)/+}$ mice *(F)* were monitored for survival.

Figure S2

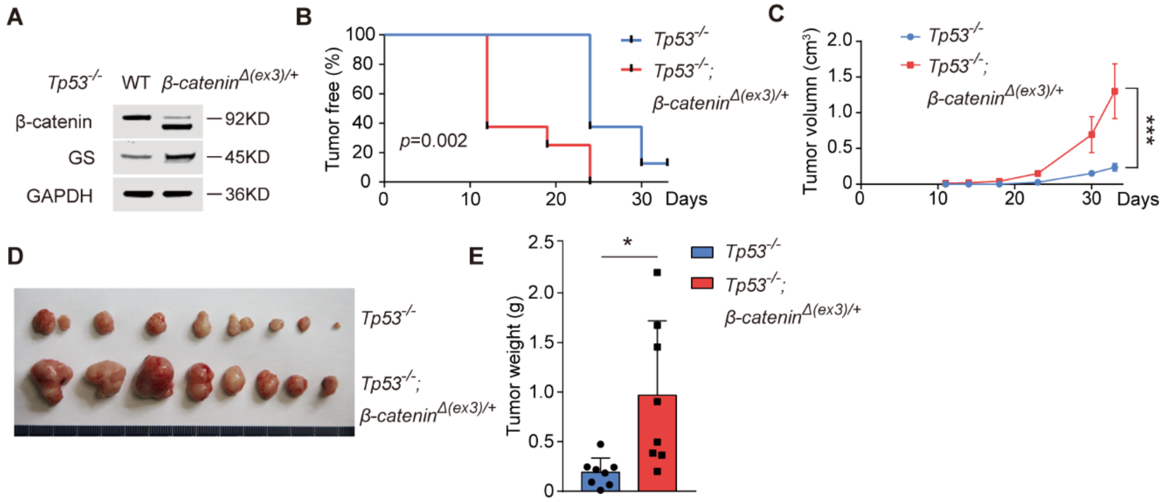

Fig. S2 β-catenin activation boosts tumorigenic potential of TP53 null MEFs.

(A) Immunoblotting of MEFs. Tumor free curves *(B)*, tumor volume curves *(C)*, tumor pictures *(D)* and tumor weight statistical analysis *(E)* of nude mice subcutaneously inoculated with wildtype and *β-catenin*$^{Δ(ex3)/+}$ MEFs (n=8 per group). *$p < 0.05$; ***$p < 0.001$. Data are shown as mean ± SD.

Figure S3

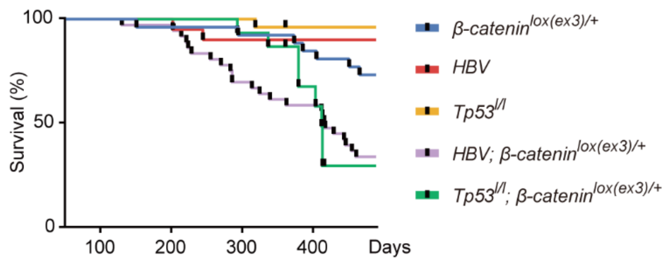

Fig. S3 Survival of Cre-adenovirus-inoculated *β-catenin*$^{lox(ex3)/+}$ mice, *HBV* mice, *Tp53*$^{l/l}$ mice, *HBV*; *β-catenin*$^{lox(ex3)/+}$ mice, and *Tp53*$^{l/l}$; *β-catenin*$^{lox(ex3)/+}$ mice.

β-catenin$^{lox(ex3)/+}$ mice (n=27), *HBV* mice (n=21), *Tp53*$^{l/l}$ mice (n=27), *HBV*; *β-catenin*$^{lox(ex3)/+}$ mice (n=39) and *Tp53*$^{l/l}$; *β-catenin*$^{lox(ex3)/+}$ mice (n=20) were injected with Cre-adenovirus via tail vein 7 weeks after birth. Survivals of these mice were monitored up to 485 days. *β-catenin*$^{lox(ex3)/+}$ mice *vs HBV* mice, p=0.0645; *β-catenin*$^{lox(ex3)/+}$ mice *vs Tp53*$^{l/l}$ mice, p=0.0538; *β-catenin*$^{lox(ex3)/+}$ mice *vs HBV*; *β-catenin*$^{lox(ex3)/+}$ mice, $p < 0.001$; *β-catenin*$^{lox(ex3)/+}$ mice *vs Tp53*$^{l/l}$; *β-catenin*$^{lox(ex3)/+}$ mice, $p < 0.001$; *HBV* mice *vs HBV*; *β-catenin*$^{lox(ex3)/+}$ mice, $p < 0.001$; *Tp53*$^{l/l}$ mice *vs Tp53*$^{l/l}$; *β-catenin*$^{lox(ex3)/+}$ mice, $p < 0.001$.

Figure S4

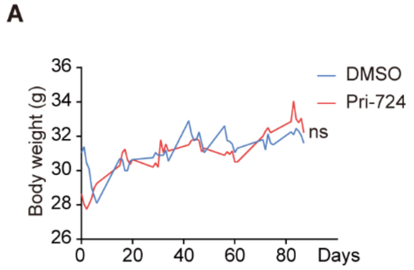

Fig. S4 Pri-724 does not affect body weight of mice.

7-week-old *HBV; β-catenin*$^{lox(ex3)/+}$ mice were first injected with *Cre*-adenovirus to cause somatic β-catenin mutation in mouse liver. These mice were then treated with DMSO or Pri-724 from 5 months to 8 months after birth. Body weights were analyzed.

Figure S5

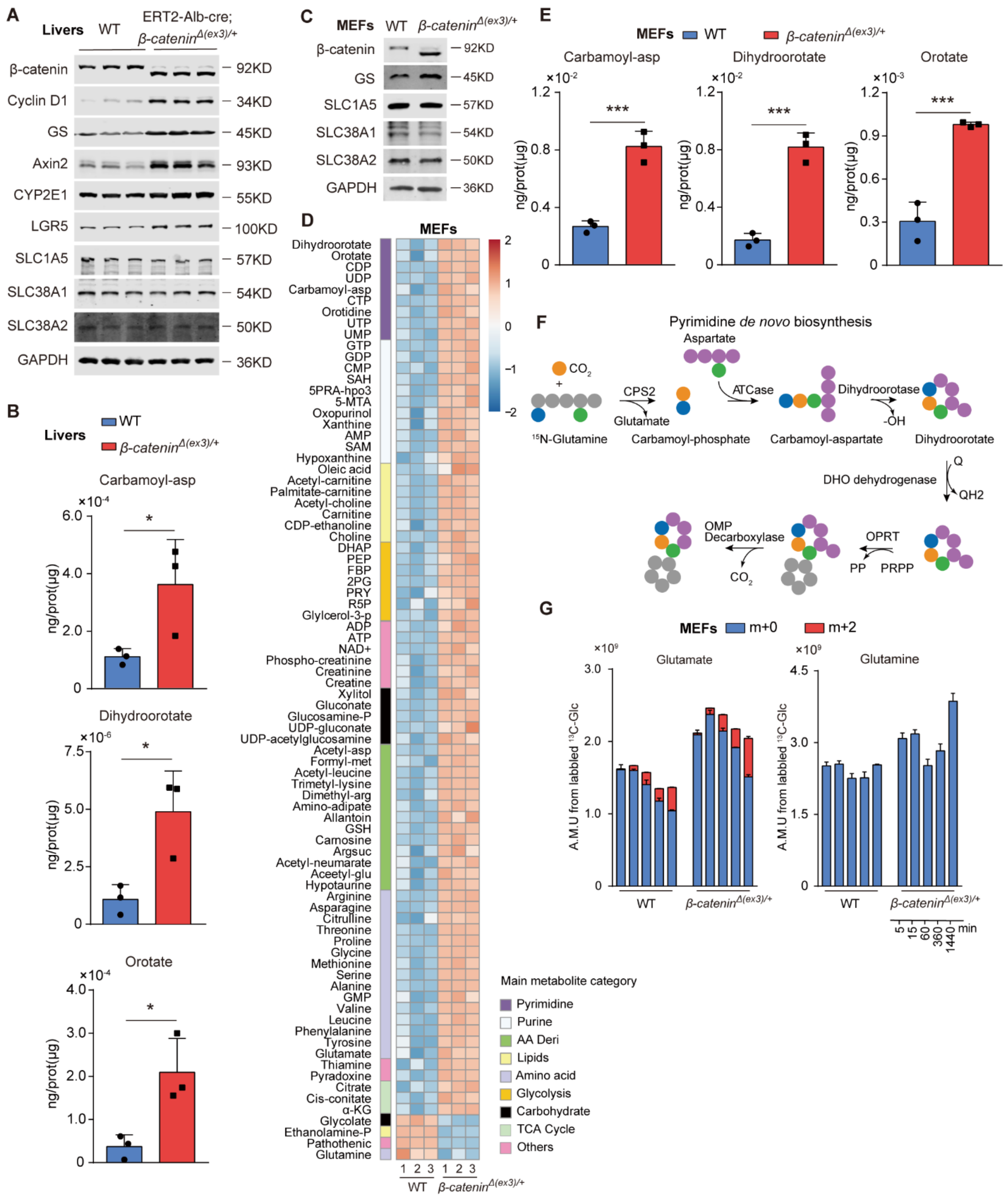

Fig. S5 Oncogenic β-catenin stimulates *de novo* pyrimidine synthesis.

(A) Immunoblotting of livers. *(B)* Steady state levels of pyrimidine biosynthesis metabolites of livers. *(C)* Immunoblotting of MEFs. *(D)* Steady-state metabolite heatmaps of MEFs. *(E)* Steady state levels of

metabolites in *β-catenin*$^{Δ(ex3)/+}$ MEFs *(F)* Schematic illustration of *de novo* pyrimidine synthesis pathway. *(G)* The incorporation flux of glutamate and glutamine from ^{13}C-glucose in MEFs at indicated time points. Data are representative of at least three independent experiments. *$p < 0.05$; ***$p < 0.001$. Analysis was performed using *t* test. Data are shown as mean ± SD.

Figure S6

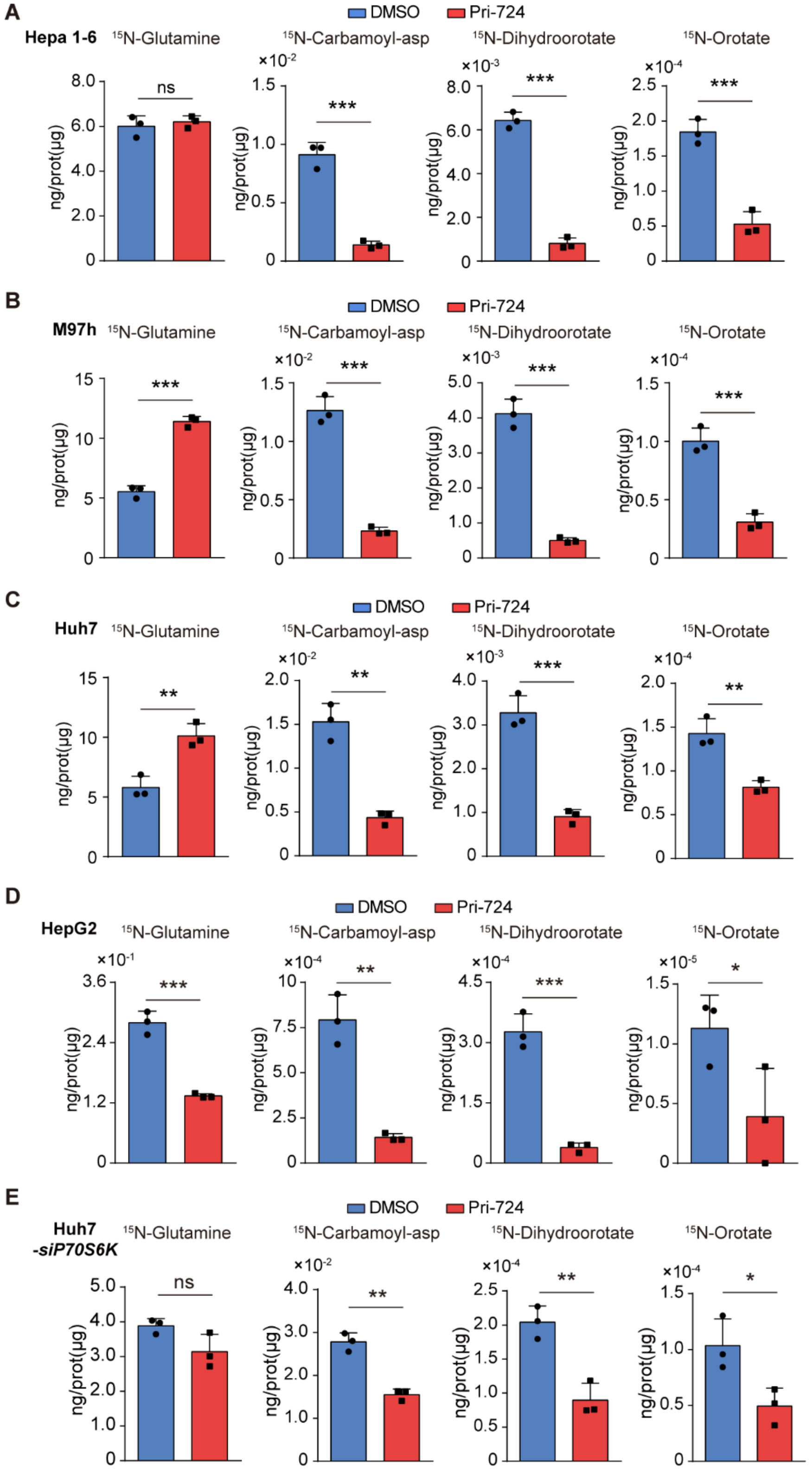

Fig. S6 Stimulation of pyrimidine synthesis by oncogenic β-catenin in various cell types.

Abundance of ^{15}N-labeled metabolites in Hepa1-6 *(A)*, M97h *(B)*, Huh7 *(C)*, HepG2 *(D)* and S6K-depleted Huh7 cells *(E)* with DMSO or Pri-724 (20μM) treatment for 24 hours and a 12-minute pulse labelling of ^{15}N-glutamine. *$p < 0.05$; ** $p < 0.01$; *** $p < 0.001$. Analysis was performed using *t* test. Data are shown as mean ± SD.

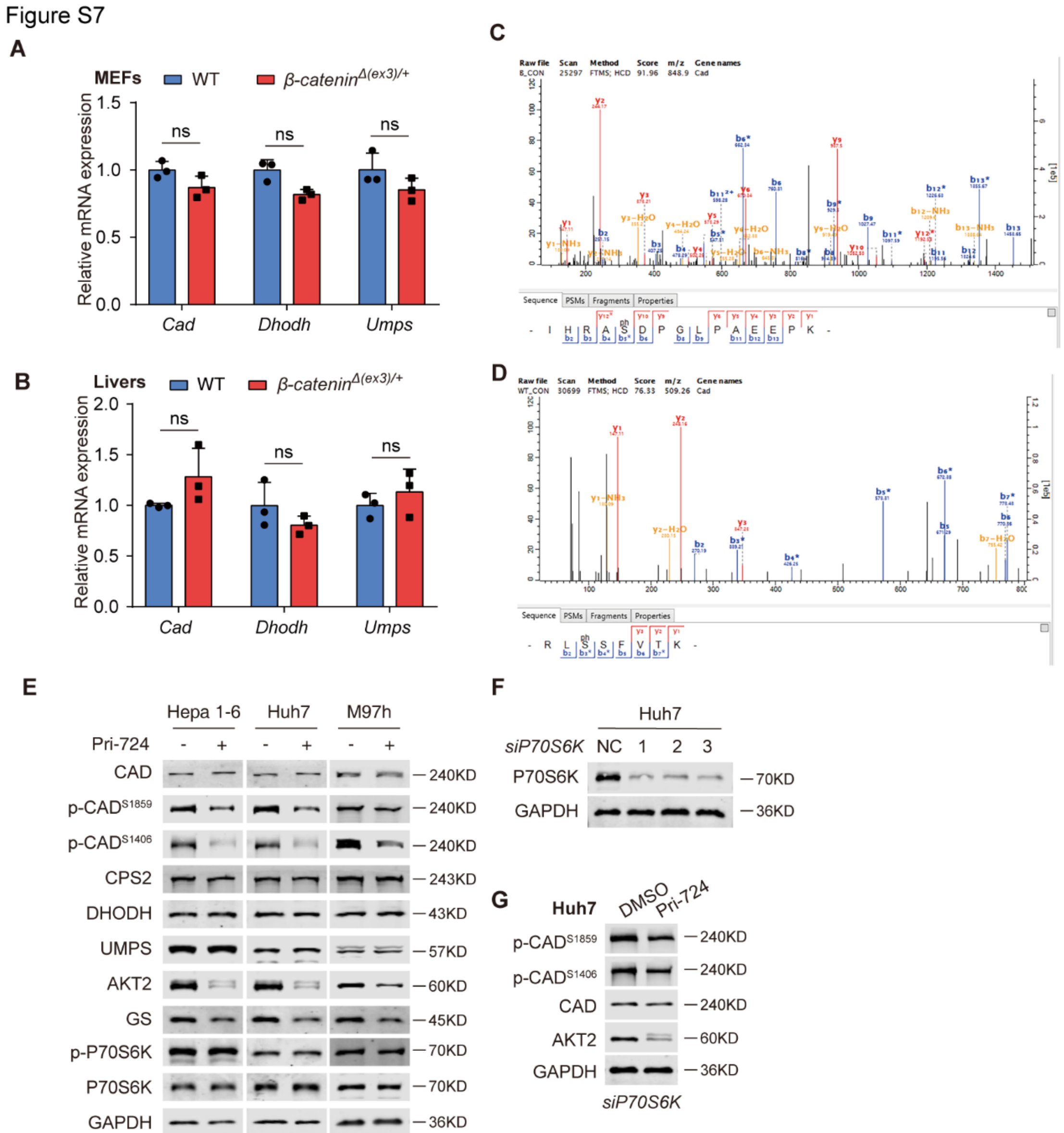

Fig. S7 Oncogenic β-catenin potentiates CAD phosphorylation and promotes *Akt2* transcription. *(A, B) Cad*, *Dhodh* and *Umps* mRNA levels of MEFs *(A)* and mouse livers *(B)* were measured by qRT-PCR and are presented as mean ± SD relative to wildtype MEFs or livers, respectively. *(C, D)* LC/MS/MS tandem mass spectrum of CAD protein from MEFs *(C)* and mouse livers *(D)*. The phosphorylation sites including S1406 and S1859 of CAD protein are shown as indicated. *(E-G)* Immunoblotting. *(E)* Hepa1-6, M97h and Huh7 cells with DMSO or Pri-724 (20μM) treatment for 24 hours; *(F)* Validation of S6K depletion. Huh7 cells were transfected with scramble or *S6K*-targeted siRNAs for 48 hours. *(G)* S6K-depleted Huh7 cells were treated with DMSO or Pri-724 (20μM) for 24 hours.

Figure S8

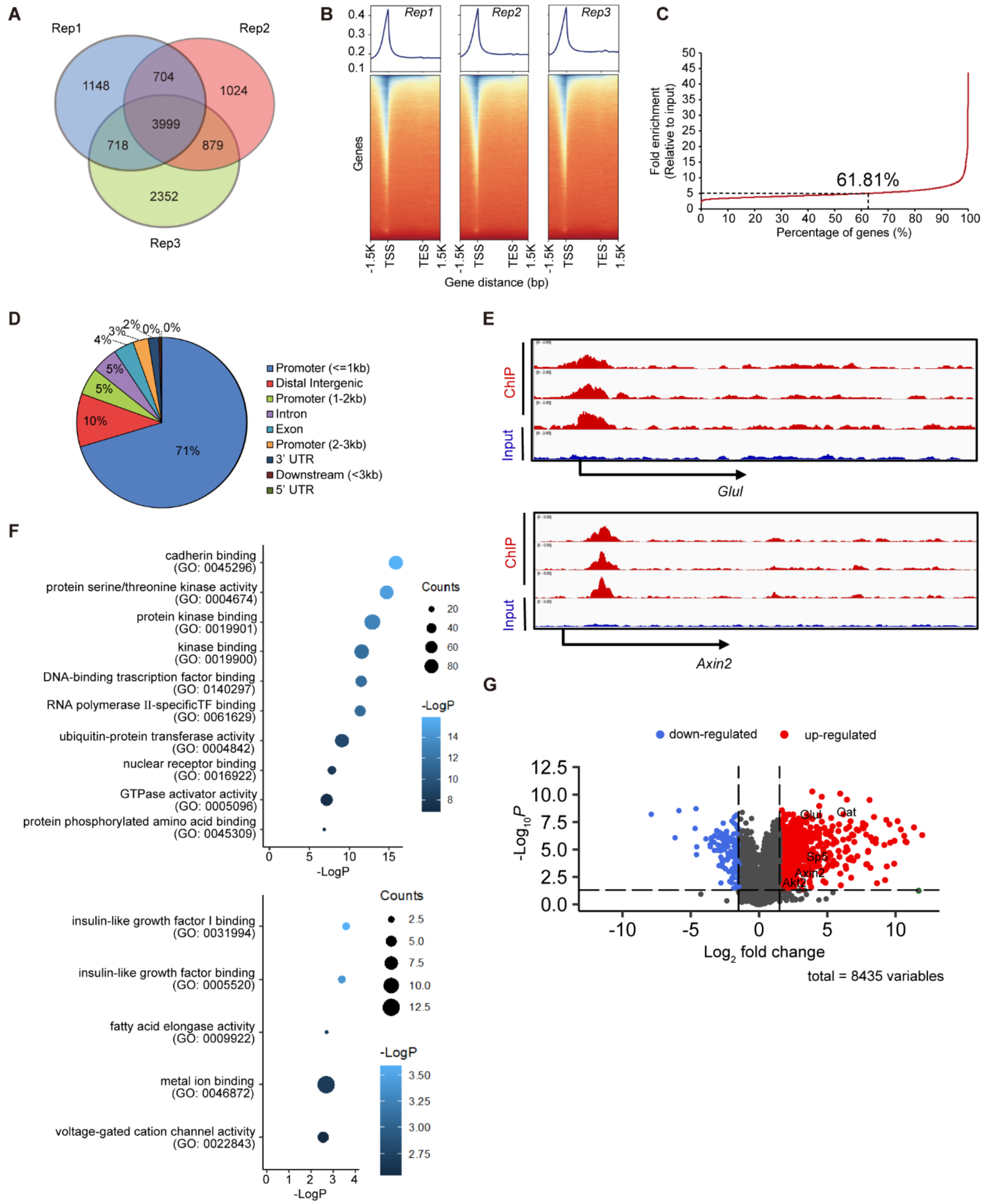

Fig. S8 Analysis of ChIP-seq and RNA-seq for mouse livers.

(*A-E*) β-catenin ChIP-seq was performed for *β-catenin*$^{\Delta(ex3)/+}$ livers. (*A*) The overlap of β-catenin-binding genes in 3 replicates (n=3999). (*B*) Upper: Metagene representation of β-catenin ChIP-seq signals. Lower:

Heat map of reproducible counts centered around a gene transcription start site for β-catenin ChIP-seq samples in mouse livers (n=3). TSS: transcription start site, TTS: transcription terminate site. (*C*) An average fold enrichment profile is shown for genes enriched>2-fold (n=387) with $p < 0.01$ and FDR < 0.01. (*D*) Binding pattern of β-catenin (n=3999). (*E*) β-catenin ChIP-seq profiles at *Glul* and *Axin2* genomic loci. (*F-G*) RNA-seq was performed for wildtype and *β-catenin*$^{\Delta(ex3)/+}$ livers. (*F*) GO annotation of upregulated genes (Upper) and down-regulated (Lower) genes. (*G*) Analysis of differentially expressed genes.

Figure S9

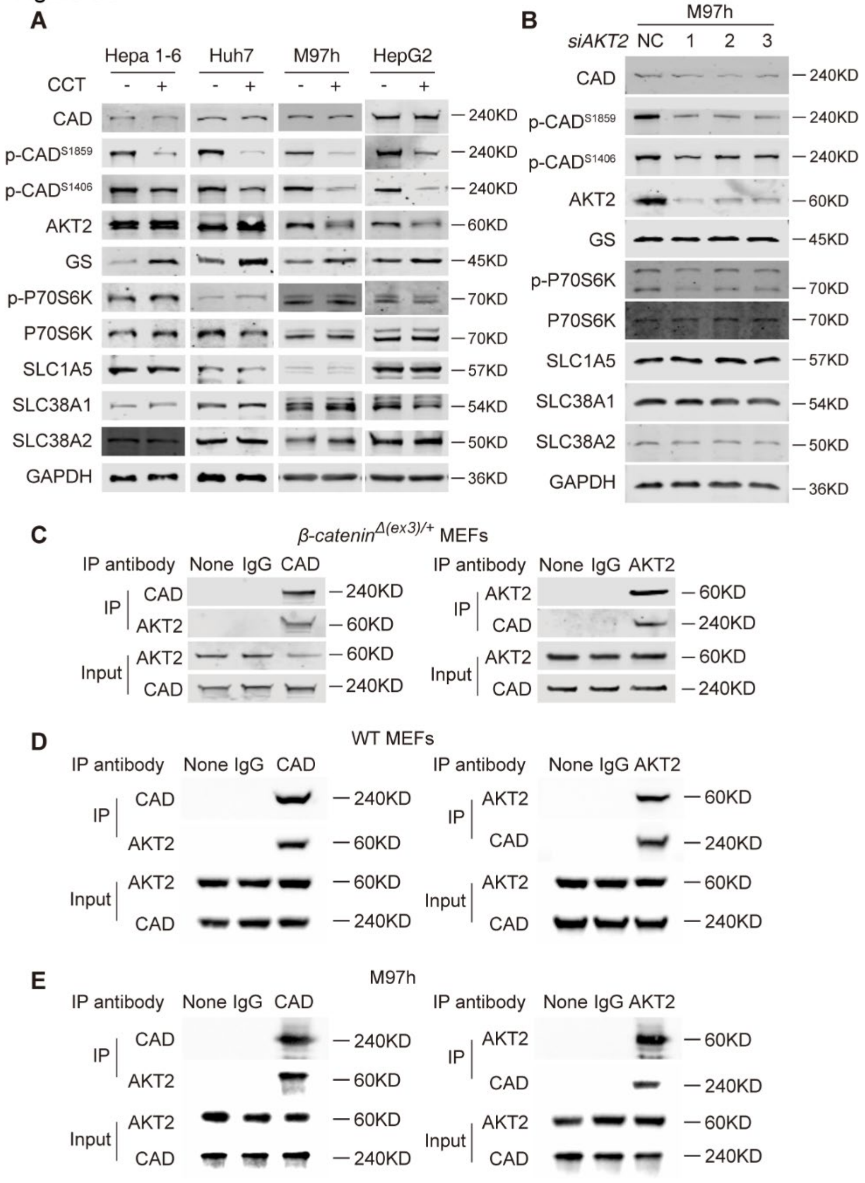

Fig. S9 CAD is a substrate of AKT2.

(*A*) Immunoblotting of Hepa 1-6, M97h, Huh7 and HepG2 cells treated with DMSO or CCT for 6 hours. (*B*) Immunoblotting of M97h cells transfected with scramble or *Akt2* targeted siRNAs. (*C-E)* Co-immunoprecipitation assays were performed using lysates from *β-catenin*$^{Δ(ex3)/+}$ MEFs (*C*), wildtype MEFs (*D*) and M97h cells (*E*).

Figure S10

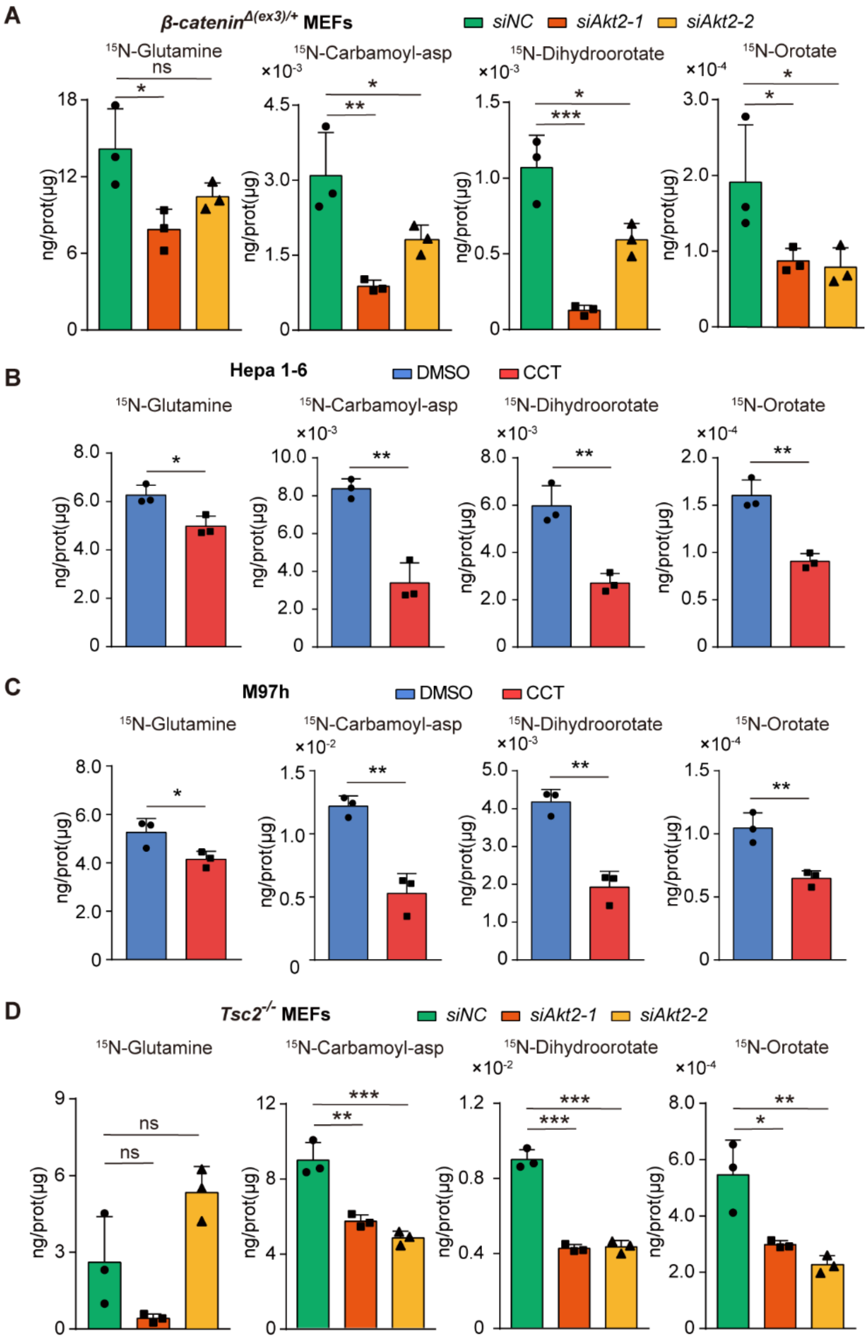

Fig. S10 AKT2 promotes *de novo* pyrimidine synthesis in various cell settings.

Abundance of ^{15}N-labeled metabolites. (*A*) 48 hours after scramble or *Akt2* siRNAs transfection, *β-catenin*$^{\Delta(ex3)/+}$ MEFs were then pulse labelled with ^{15}N-glutamine for 12 minutes. (*B-D*) Hepa 1-6 *(B)*, M97h *(C)* and Huh7 cells *(D)* were treated with vehicle or CCT (5μM) for 6 hours and then pulse labelled with ^{15}N-glutamine for 12 minutes. *(E)* 48 hours after scramble or *Akt2* siRNAs transfection, *Tsc2*$^{-/-}$ MEFs were pulse

labelled with ^{15}N-glutamine for 12 minutes. *p < 0.05; **p < 0.01; ***p < 0.001. Analysis was performed using t test. Data are shown as mean ± SD.

Figure S11

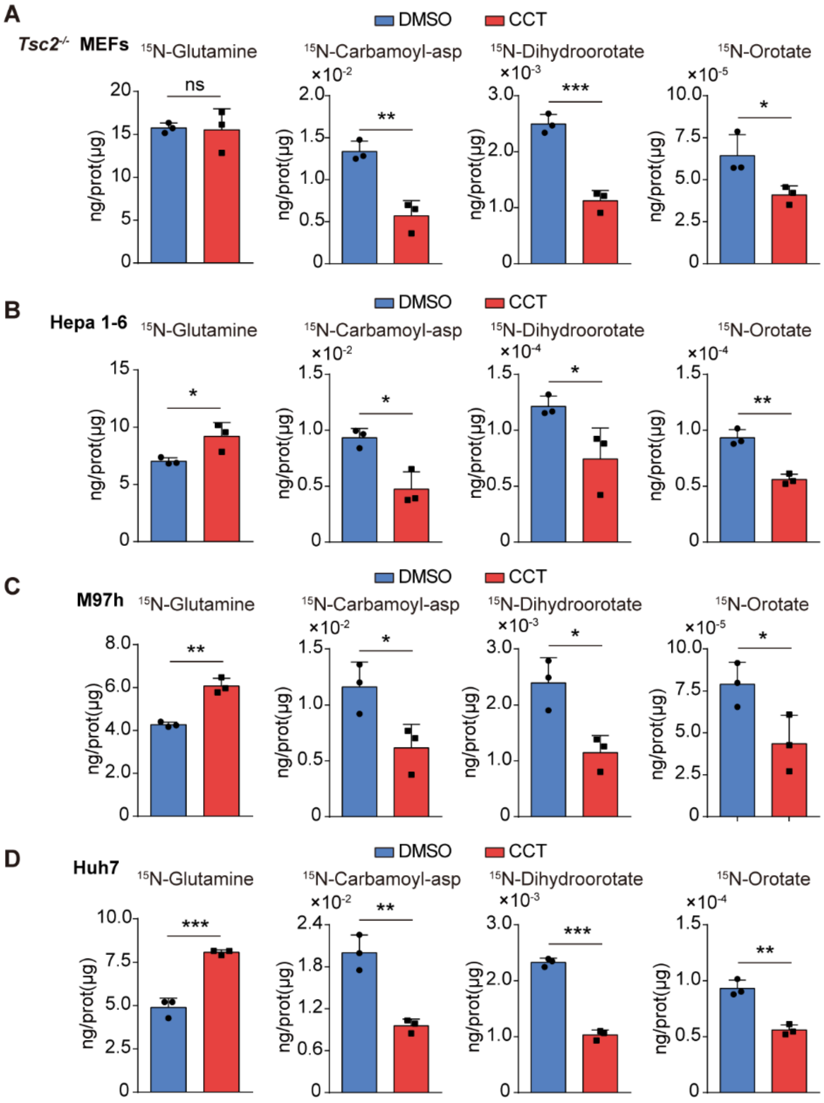

Fig. S11 Interferences of AKT2 abolish pyrimidine synthesis in glutamine-abundant conditions. Abundance of ^{15}N-labeled metabolites. *Tsc2*$^{-/-}$ MEFs (*A*), Hepa 1-6 cells (*B*), M97h cells (*C*) and Huh7 cells (*D*) were treated with DMSO or CCT (5μM) for 6 hours and a 12-minute pulse labelling of ^{15}N-labeled glutamine. Excessive glutamine (6 mM) was provided for CCT-treated cells and standard dose of glutamine (2 mM) was provided for DMSO-treated cells. *$p < 0.05$; **$p < 0.01$; ***$p < 0.001$. Analysis was performed using *t* test. Data are shown as mean ± SD.

Figure S12

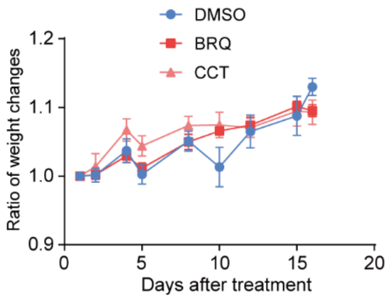

Fig. S12 BRQ or CCT treatment does not affect body weight of nude mice.

Body weight changes of nude mice. *β-catenin*$^{\Delta(ex3)/+}$ MEFs bearing nude mice were treated with DMSO, BRQ or CCT.

Figure S13

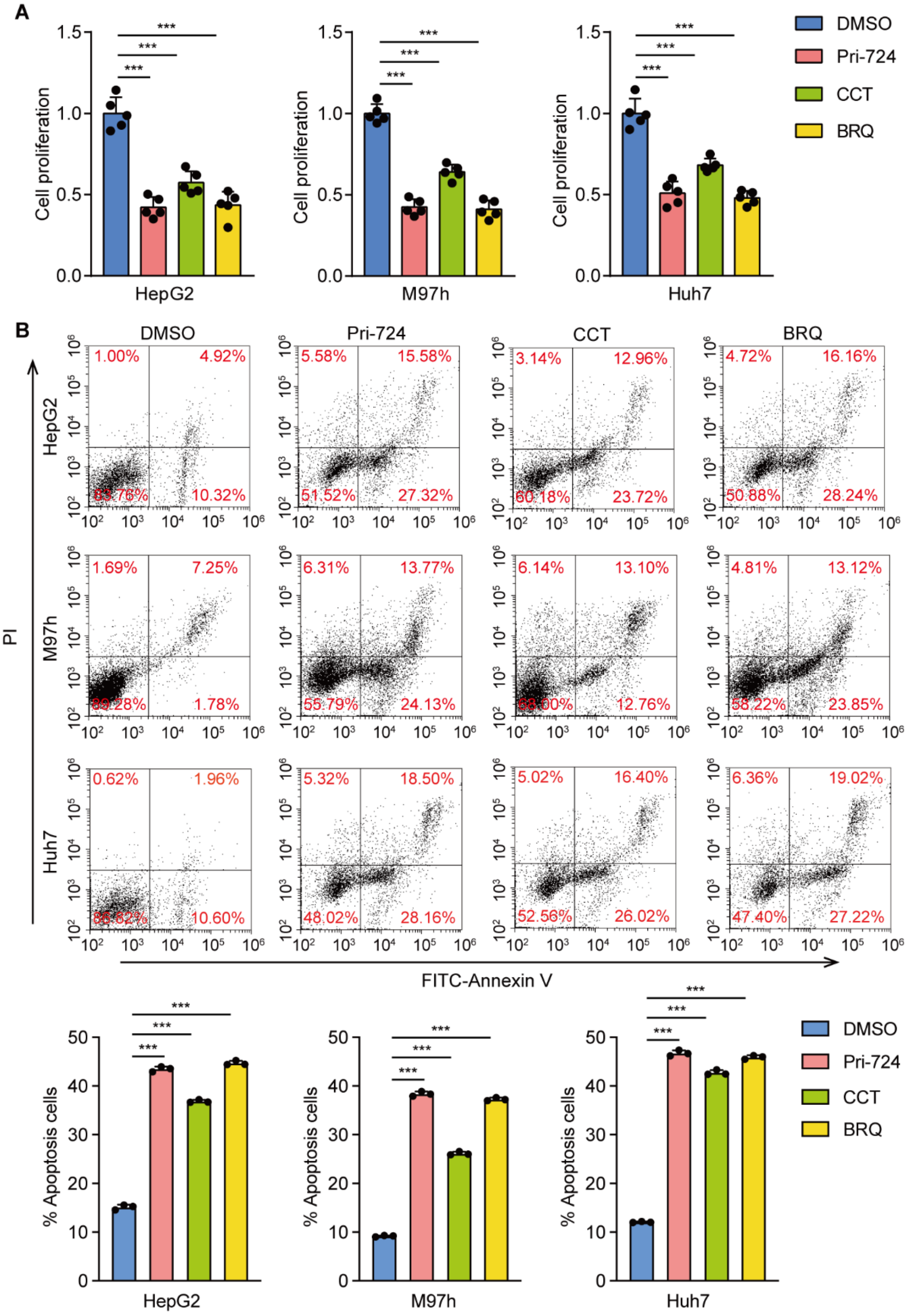
A
Cell proliferation
HepG2
M97h
Huh7
DMSO
Pri-724
CCT
BRQ
B
DMSO
Pri-724
CCT
BRQ
HepG2
M97h
Huh7
PI
FITC-Annexin V
1.00%
4.92%
83.76%
10.32%
5.58%
15.58%
51.52%
27.32%
3.14%
12.96%
60.18%
23.72%
4.72%
16.16%
50.88%
28.24%
1.69%
7.25%
89.28%
1.78%
6.31%
13.77%
55.79%
24.13%
6.14%
13.10%
68.00%
12.76%
4.81%
13.12%
58.22%
23.85%
0.62%
1.96%
86.82%
10.60%
5.32%
18.50%
48.02%
28.16%
5.02%
16.40%
52.56%
26.02%
6.36%
19.02%
47.40%
27.22%
% Apoptosis cells

Fig. S13 Suppressing β-catenin/AKT/pyrimidine synthesis inhibits proliferation and induces apoptosis of liver cancer cells.

HepG2, M97h and Huh7 cells were treated with DMSO, Pri-724 (20μM), CCT (5μM) or BRQ (5μM) for 48 hours. Cell proliferation *(A)* was checked by CCK8 assay and cell apoptosis was assessed by flow cytometry*(B)*. Data are shown as mean ± SD. ***$p < 0.001$.

Figure S14

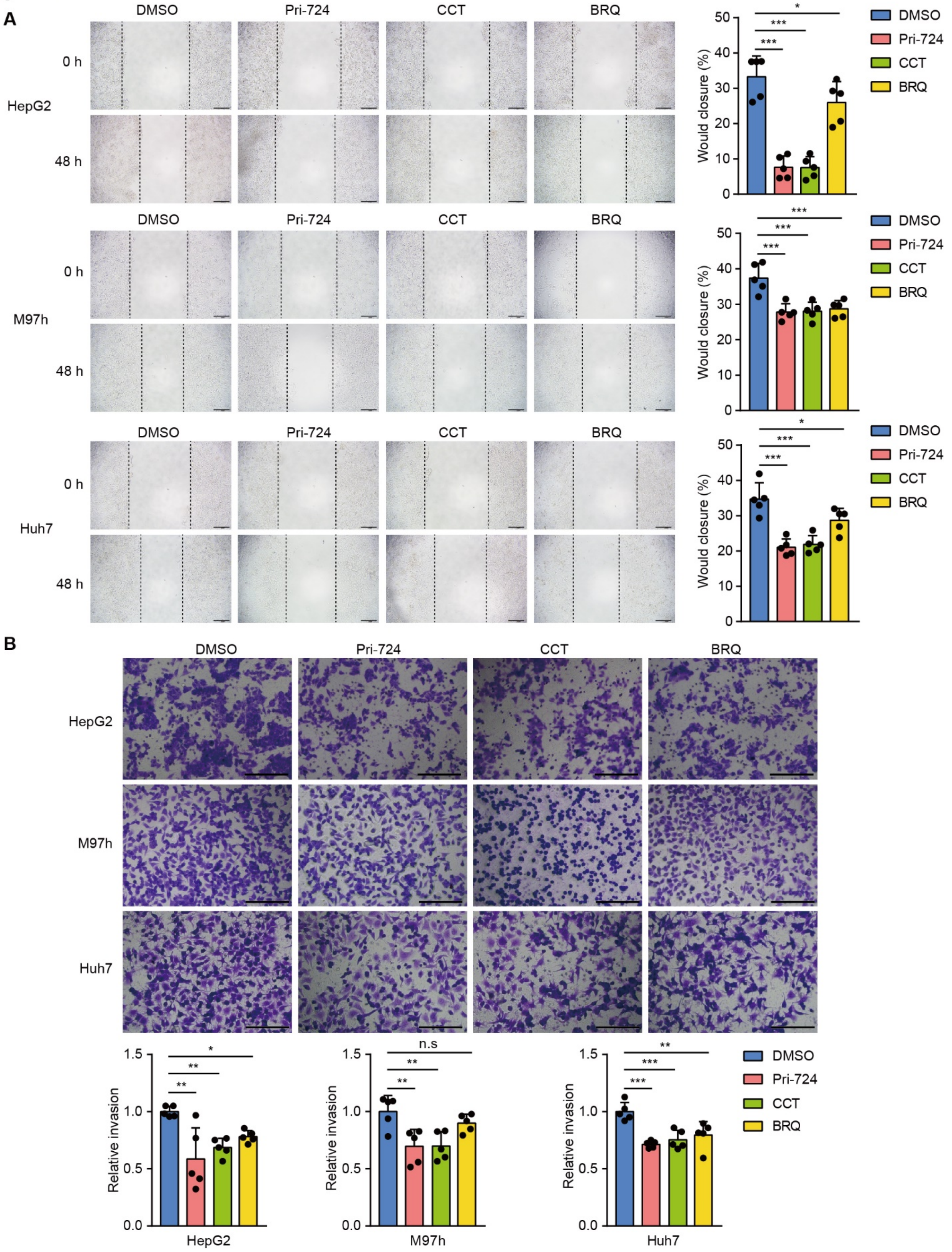
A
DMSO
Pri-724
CCT
BRQ
0 h
48 h
HepG2
M97h
Huh7
Would closure (%)
B
Relative invasion
n.s
HepG2
M97h
Huh7

Fig. S14 Effect of suppressing β-catenin/AKT/pyrimidine synthesis on migration and invasion of liver cancer cells.

HepG2, M97h and Huh7 cells were treated with DMSO, Pri-724 (20μM), CCT (5μM) or BRQ (5μM). Cell migration was examined by wound scratch assay (48 h) *(A)* and cell invasion was evaluated by transwell assay (24 h) *(B)*. Data are shown as mean ± SD. $^*p < 0.05$, $^{**}p < 0.01$, $^{***}p < 0.001$.

Figure S15

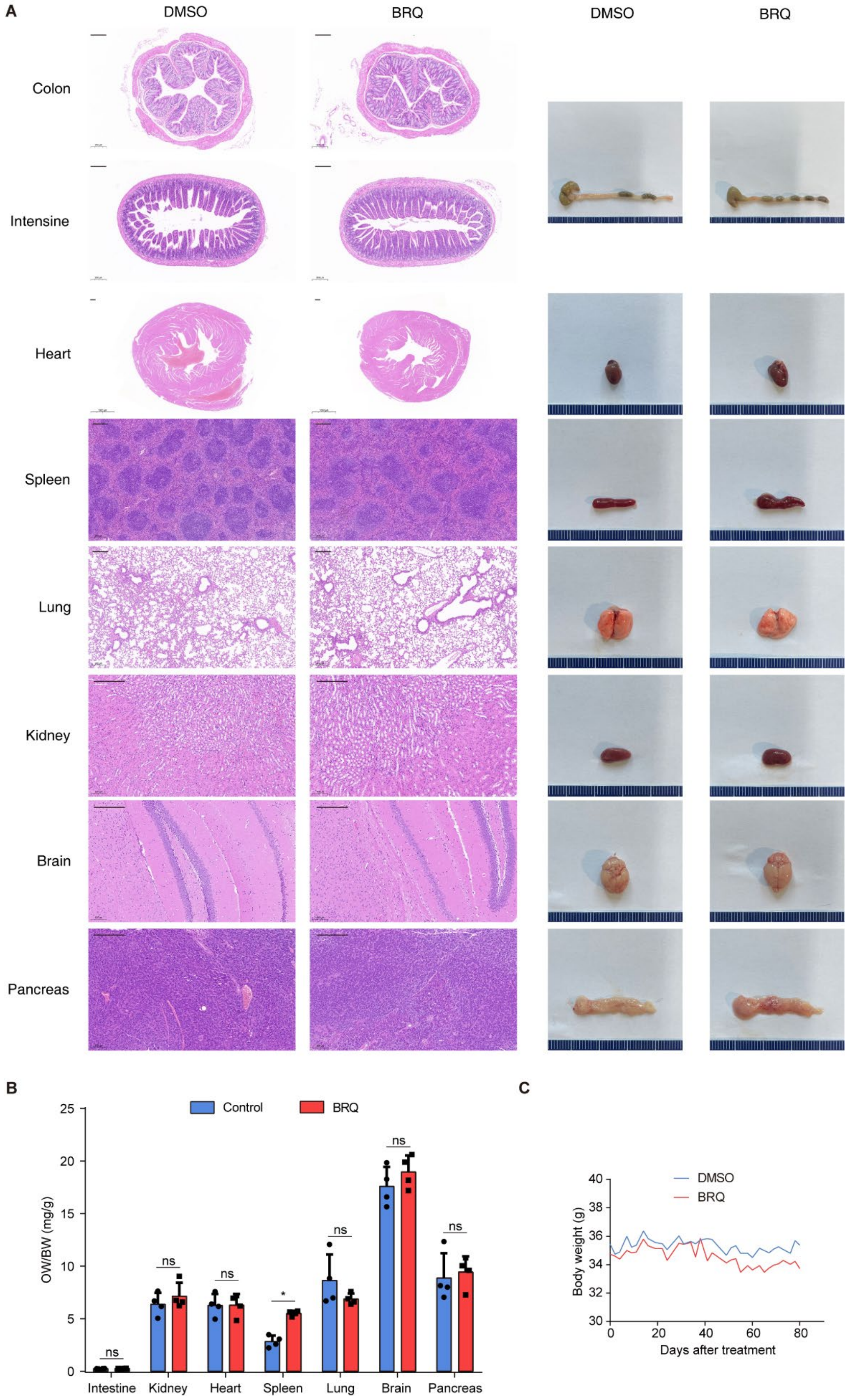
A
DMSO
BRQ
DMSO
BRQ
Colon
Intensine
Heart
Spleen
Lung
Kidney
Brain
Pancreas
B
Control
BRQ
OW/BW (mg/g)
25
20
15
10
5
0
ns
ns
ns
*
ns
ns
ns
Intestine
Kidney
Heart
Spleen
Lung
Brain
Pancreas
C
DMSO
BRQ
Body weight (g)
40
38
36
34
32
30
0
20
40
60
80
Days after treatment

Fig. S15 BRQ has minimal toxicity for mice.

7- week-old *HBV; β-catenin*$^{lox(ex3)/+}$mice were injected via tail vein with *Cre*-adenoviruses. DMSO or BRQ treatment was started when mice were 7-month-old. Mice were sacrificed 3 months later. (*A*) H&E staining and pictures of the organs. (*B*) Ratios of organ weight to body weight. (*C*) Body weight changes of mice. Analysis was performed using *t* test. Data are shown as mean ± SD.

Table S1. Tumor formation in *β-catenin*$^{lox(ex3)/+}$ mice.

No	Benign	Malignant
1	-	-
2	-	-
3	-	-
4	-	-
5	-	-
6	-	-
7	-	-
8	-	+
9	-	+
10	+	+
11	-	+
12	+	-
13	+	+
14	+	+
15	-	+
16	+	+
17	-	+
18	+	+
19	+	+
20	+	+
21	+	-
22	-	+
23	+	-
24	-	+
25	+	+
26	-	+
**Percentage**	**42.3%**	**61.5%**

Liver tumor formation in each 13-month-old *β-catenin*$^{lox(ex3)/+}$ mouse which had tail vein injection of *Cre*-adenoviruses at age of 7 weeks. "+" indicates visible tumors on the surface of livers, while "-" indicates no visible tumors.

Table S2. Analysis of HCC genomic sequencing data from various studies.

	Total cases	*CTNNB1* mutation	*TP53* mutation	*CTNNB1* plus *TP53* mutation	*CTNNB1* plus *TP53* mutation/*CTNNB1* mutation (%)	*CTNNB1* plus *TP53* mutation/*TP53* mutation (%)
**Gao, Q. et al. Cell, 2019**	159	26	93	12	12/26 (46%)	12/93 (13%)
**Ho, D.W.H. *et al.* Gut, 2017**	95	15	47	8	8/15 (53%)	8/47 (17%)
**Wang, S. *et al.* Hepatobiliary Surg Nutr, 2021**	168	38	94	19	19/38 (50%)	19/94 (20%)
**Zhang, W. et al. Gastroenterology, 2017**	49	10	41	5	5/10 (50%)	5/41 (12%)

Table S3. Oncogenic β-catenin stimulates phosphorylation of S1406 and S1859 in CAD.

Sample Name	Sample Type	Protein accession	Position	Mut/WT Ratio	Regulated Type	Amino acid	Gene name	Localization probability	PEP	Score	Modified sequence	Charge	MS/MS Count
**Mut/WT**	MEFs	B2RQC6	1859	1.368	Up	S	Cad	1	3.9069E-24	169.43	IHRAS(1)DPGLPAEEPK	2	1
**Mut/WT**	MEFs	B2RQC6	1406	2.851	Up	S	Cad	0.887448	0.000305255	103.76	RLS(0.887)S(0.113)FVTK	2	3
**Mut/WT**	Livers	B2RQC6	1859	1.718	Up	S	Cad	1	0.000467012	91.964	IHRAS(1)DPGLPAEEPK	12	
**Mut/WT**	Livers	B2RQC6	1406	2.792	Up	S	Cad	0.82252	0.00157649	76.332	RLS(0.823)S(0.177)FVTK	5	
**Pri/Con**	MEFs	B2RQC6	1859	0.556	Down	S	Cad	1	0.000467012	91.964	IHRAS(1)DPGLPAEEPK	3	12
**Pri/Con**	MEFs	B2RQC6	1406	0.647	Down	S	Cad	0.82252	0.00157649	76.332	RLS(0.823)S(0.177)FVTK	2	5

LC/MS spectrum analysis of the peptides IHRApSDPGLPAEEPK and RLpSSFVTK containing the phosphorylation sites of S1859 and S1406 from CAD protein in MEFs and livers.

Table S4. The docking score of peptides with AKT2.

Peptide	Receptor	Docking score (kcal/mol)
RRLSSFV	AKT2	-9.41
HRASDPG	AKT2	-8.74

Docking score of AKT2 and RRLSSFV (S1406) or HRASDPG (S1859) of CAD protein. More negative score suggests better binding of the peptide with protein.

Table S5. The contact list between RRLSSFV and AKT2.

Chain 1	Residue	Chain 2	Residue	Interaction type
RRLSSFV	Arg1	AKT2	Glu236	Salt bridge
RRLSSFV	Arg2	AKT2	Glu279	Salt bridge
RRLSSFV	Arg2	AKT2	Glu315	Salt bridge
RRLSSFV	Ser4	AKT2	Mn^{2+}	metal contact
RRLSSFV	Ser5	AKT2	Mn^{2+}	metal contact
RRLSSFV	Val7	AKT2	Glu193	Hydrogen bond interaction
RRLSSFV	Ser4	AKT2	ANP	Hydrogen bond interaction
RRLSSFV	Ser5	AKT2	ANP	Hydrogen bond interaction

Table S6. The contact list between HRASDPG and AKT2.

Chain 1	Residue	Chain 2	Residue	Interaction type
HRASDPG	Arg2	AKT2	Glu236	Salt bridge
HRASDPG	Arg2	AKT2	Asp440	Salt bridge
HRASDPG	Asp5	AKT2	Lys277	Salt bridge
HRASDPG	His1	AKT2	Glu279	Hydrogen bond interaction
HRASDPG	His1	AKT2	Glu442	Hydrogen bond interaction
HRASDPG	Arg2	AKT2	Tyr438	Hydrogen bond interaction
HRASDPG	Pro6	AKT2	His355	Hydrogen bond interaction
HRASDPG	Ser4	AKT2	ANP	Hydrogen bond interaction
HRASDPG	Arg2	AKT2	ANP	Hydrogen bond interaction

SI References

1. P. Gade, D. V. Kalvakolanu, Chromatin immunoprecipitation assay as a tool for analyzing transcription factor activity. *Methods Mol Biol* **809**, 85-104 (2012).
2. B. Langmead, S. L. Salzberg, Fast gapped-read alignment with Bowtie 2. *Nat Methods* **9**, 357-359 (2012).
3. Y. Zhang *et al.*, Model-based analysis of ChIP-Seq (MACS). *Genome Biol* **9**, R137 (2008).
4. T. Liu, Use model-based Analysis of ChIP-Seq (MACS) to analyze short reads generated by sequencing protein-DNA interactions in embryonic stem cells. *Methods Mol Biol* **1150**, 81-95 (2014).
5. G. Yu, L. G. Wang, Q. Y. He, ChIPseeker: an R/Bioconductor package for ChIP peak annotation, comparison and visualization. *Bioinformatics* **31**, 2382-2383 (2015).
6. F. Ramírez *et al.*, deepTools2: a next generation web server for deep-sequencing data analysis. *Nucleic Acids Res* **44**, W160-165 (2016).
7. X. Gai *et al.*, mTOR/miR-145-regulated exosomal GOLM1 promotes hepatocellular carcinoma through augmented GSK-3beta/MMPs. *J Genet Genomics* **46**, 235-245 (2019).
